# Interferons and interferon-related pathways in heart disease

**DOI:** 10.3389/fcvm.2024.1357343

**Published:** 2024-04-11

**Authors:** Duc Tin Tran, Sri Nagarjun Batchu, Andrew Advani

**Affiliations:** Keenan Research Centre for Biomedical Science and Li Ka Shing Knowledge Institute, St. Michael’s Hospital, Toronto, ON, Canada

**Keywords:** interferon, interferon-stimulated gene, heart failure, myocardial infarction, cGAS-STING, interferon regulatory factor, type I interferon (IFN), interferon gamma (IFN-γ)

## Abstract

Interferons (IFNs) and IFN-related pathways play key roles in the defence against microbial infection. However, these processes may also be activated during the pathogenesis of non-infectious diseases, where they may contribute to organ injury, or function in a compensatory manner. In this review, we explore the roles of IFNs and IFN-related pathways in heart disease. We consider the cardiac effects of type I IFNs and IFN-stimulated genes (ISGs); the emerging role of the cyclic GMP-AMP synthase (cGAS)-stimulator of interferon genes (STING) pathway; the seemingly paradoxical effects of the type II IFN, IFN-γ; and the varied actions of the interferon regulatory factor (IRF) family of transcription factors. Recombinant IFNs and small molecule inhibitors of mediators of IFN receptor signaling are already employed in the clinic for the treatment of some autoimmune diseases, infections, and cancers. There has also been renewed interest in IFNs and IFN-related pathways because of their involvement in SARS-CoV-2 infection, and because of the relatively recent emergence of cGAS-STING as a pattern recognition receptor-activated pathway. Whether these advances will ultimately result in improvements in the care of those experiencing heart disease remains to be determined.

## Introduction

It is over sixty five years since a substance that interferes with viral replication in host cells, termed “interferon”, was first reported by Isaacs and Lindemann (1). Since that first description, our knowledge of interferons (IFNs), their upstream regulators, downstream effects, and related regulatory factors continues to expand well beyond the field of virology. IFNs and IFN-related pathways are emerging as critical determinants of the pathogenesis of heart disease, or indeed on occasion protection against it. In this review article, we discuss the emerging state-of-the-art of IFNs and IFN-related pathways in the heart focusing on type I IFNs, the type I IFN response, and interferon-stimulated genes (ISGs); the related upstream cyclic GMP-AMP synthase (cGAS)- stimulator of interferon genes (STING) signaling pathway; the seemingly paradoxical actions of the type II IFN, IFN-γ; and the interferon regulatory factor (IRF) family of transcription factors.

### The IFN family, IFN function and IFN induction

**Figure 1:**
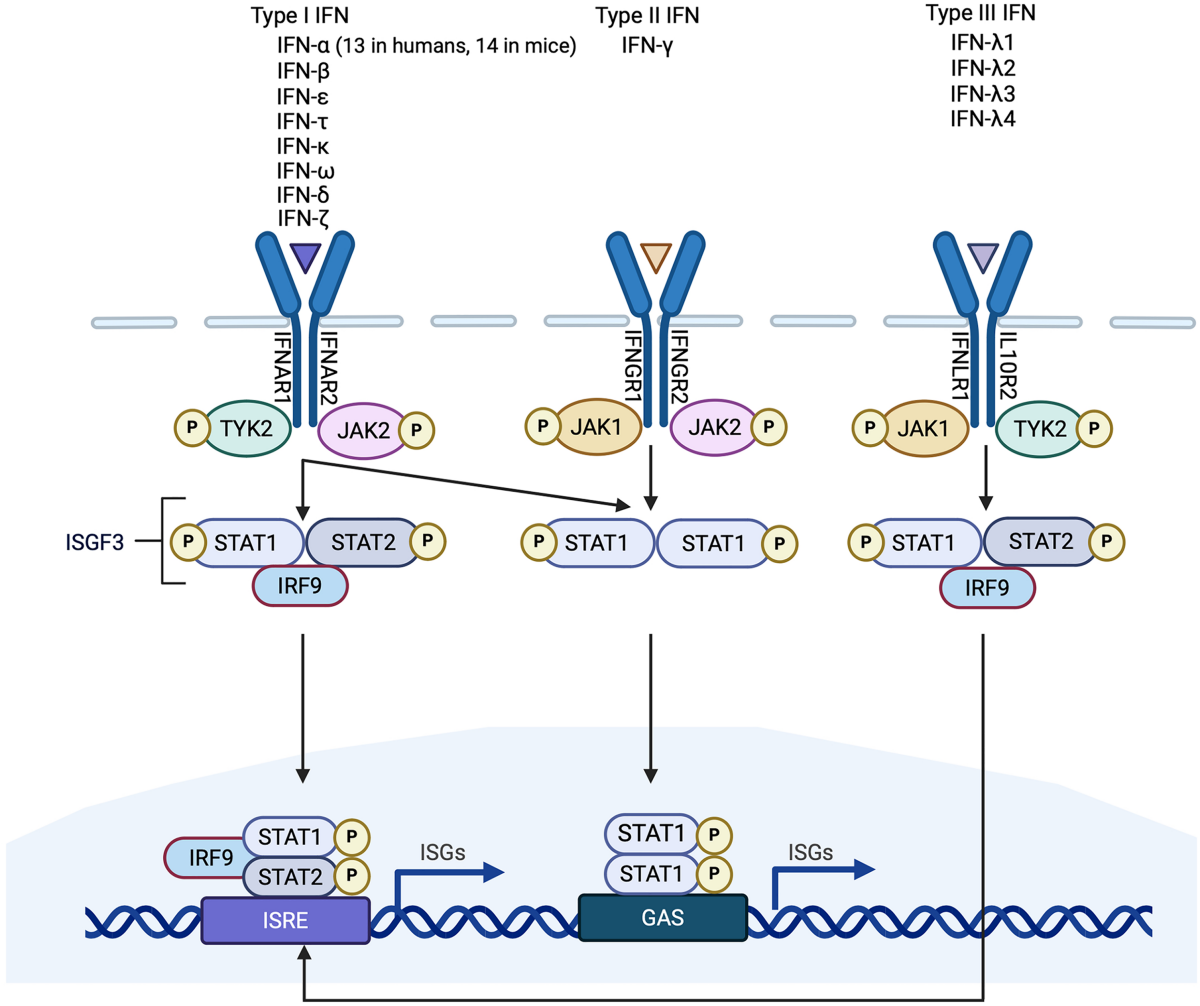
Simplified schematic of interferon (IFN) signaling pathways. Type 1 IFNs are the largest of the three classes of IFN. They signal through IFNAR1/2 which activates JAK/STAT signaling. STAT1/2 phosphorylation causes the release of STATs from IFNAR and the formation of a complex containing phosphorylated STAT1/2 and IRF9, called interferon-stimulated gene factor 3 (ISGF3). ISGF3 binds to a promoter sequence called interferon-stimulated response element (ISRE) and induces interferon stimulated genes (ISGs). There is only one Type II IFN (IFN-γ). It binds to IFNGR1/2 to activate JAK/STAT signaling and the formation of phosphorylated STAT1 homodimers which bind to IFN-γ-activated sites (GAS) elements in gene promoters. Type I IFNs can also induce STAT1 homodimerization. Type III IFNs signal through IFNLR1/IL10R2 and, like type I IFNs, induce the formation of an ISGF3 complex. Non-canonical signaling pathways are not shown.

IFNs belong to the Class II cytokine family, a group of α-helical cytokines with modest sequence homology but structural similarity (2). The IFN family itself is made up of three classes, distinguished from one another according to the type of receptor that they bind to: type I IFNs, type II IFN (of which there is only one), and type III IFNs (Figure 1). The type I IFNs consist of five α-helices (3), and bind to a ubiquitously expressed heterodimeric receptor that is made of two chains called IFNAR1 and IFNAR2. Within the type I IFN class, and the most extensively studied of the IFNs, are IFN-α and IFN-β. Thirteen genes encode for human IFN-α (in mice there are 14), whereas IFN-β is encoded by a single gene (3, 4). Other type I IFNs are less well characterized. These include IFN-ε, IFN-τ, IFN-κ, IFN-ω, IFN-δ, and IFN-ξ (3). There is only one member of the type II IFN class, IFN-γ. Whereas type I IFNs are monomers, IFN-γ is an intercalated dimer (3). IFN-γ is structurally unrelated to the type I IFNs and it binds to a different receptor, which is made up of IFNGR1 and IFNGR2 subunits (5). The type III IFNs [made up of IFN-λ1, IFN-λ2 and IFN-λ3, also called interleukin-28A (IL-28A), IL-28B and IL-29 respectively, and IFN-λ4] are structurally related to type I IFNs and also to IL-10 (5). They bind to a heterodimeric receptor made up of IL10R2 and IFNLR1 subunits (5), and they are the least characterized IFN class.

Almost all cells can be induced to express type I IFNs, although the main sources of type I IFNs are innate immune cells (6). IFN-γ expression, in contrast, is more restricted, being primarily expressed by T cells [CD8+ cytotoxic T cells and T helper type 1 (Th1) cells] and natural killer (NK) cells (5). However, most cells express IFNGR and therefore most cells respond to IFN-γ (7). IFN-γ can induce the expression of genes that prime the type I IFN response, and type I IFNs can potentiate IFN-γ signaling (7, 8). Type III IFNs, like type I IFNs, can also be expressed by most cells, although they mainly act at epithelial surfaces (5, 9, 10).

**Figure 2:**
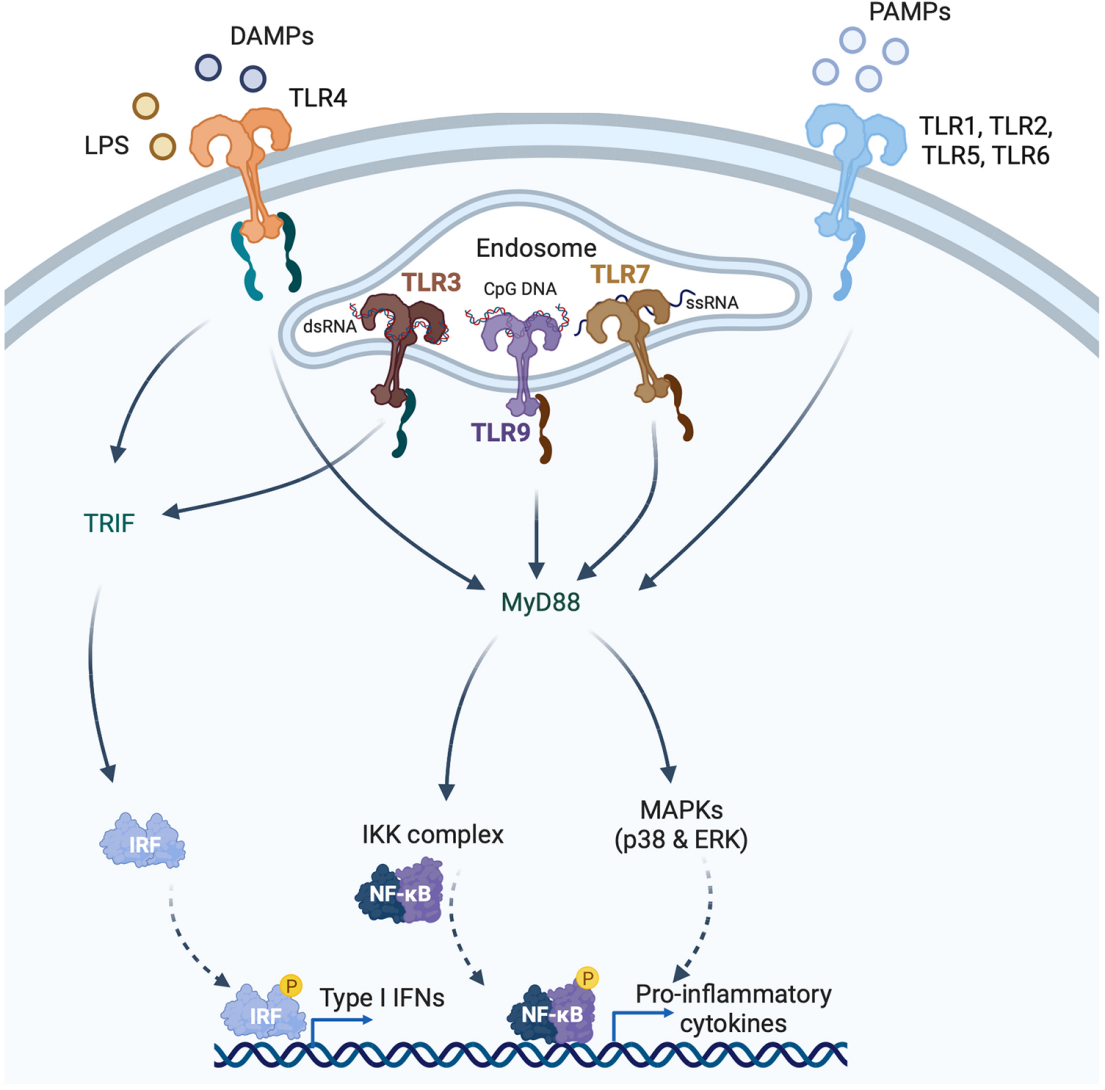
Induction of type I interferons (IFNs) by pattern recognition receptors (PRRs). PRRs recognize pathogen-associated molecular patterns (PAMPs) and damage-associated molecular patterns (DAMPs) that are either intracellular or extracellular. Toll like receptors (TLRs) are membrane bound PRRs and major inducers of the IFN response. TLR4 is expressed on the cell surface and recognizes bacterial lipopolysaccharide (LPS) and endogenous DAMPs. TLR1, 2, 5 and 6 recognize PAMPs. TLR3 is localized to endosomes, and recognizes viral double-stranded RNA (dsRNA), whereas TLR7 recognizes single-stranded RNA, and TLR9 recognizes CpG DNA. TLRs induce signaling via either the myeloid differentiation primary response 88 (MyD88)-dependent or TIR domain-containing adaptor inducing IFNβ (TRIF)-dependent pathways. TLR3 and TLR4 signal through the TRIF-dependent pathway, and TLR4 can also signal through the MyD88-dependent pathway. MyD88-signaling involves mitogen-activated protein kinase (MAPKs) and nuclear factor κ-light chain enhancer of activated B cells (NFκB), whereas TRIF-signaling is mediated through IRF3 phosphorylation.

The primary function of the IFNs is in the host defence against microbial infection. IFN gene expression is induced by the binding of pattern recognition receptors (PRRs) to pathogen-associated molecular patterns (PAMPs), which are molecules unique to microbes such as viruses or bacteria [e.g., nucleic acids, bacterial endotoxin (lipopolysaccharide, LPS), certain glycoproteins, bacterial peptides, and fungal glucans] (Figure 2). PRRs, however, can also be activated by endogenous molecules that are released by damaged or dying host cells. These endogenous molecules are called damage-associated molecular patterns (DAMPs). Outside of the setting of viral myocarditis or other microbial infections, induction of the IFN response in heart disease is mediated by DAMPs. PRRs can sense PAMPs (or DAMPs) that are either outside the cell (through membrane bound PRRs) or inside the cell (through cytoplasmic PRRs). Membrane bound PRRs include Toll like receptors (TLRs) and C-type lectin receptors (CLRs). TLRs are major inducers of the IFN response. TLRs can be present on the cell membrane (mediating extracellular signaling) or on the membrane of endosomes (mediating intracellular signaling) (Figure 2). For instance, TLR3 is localized to endosomes, and recognizes viral double-stranded RNA (dsRNA), small interfering RNAs (siRNAs) and host RNAs derived from damaged cells (11). TLR4, on the other hand, is expressed on the cell surface and recognizes LPS derived from invading bacteria, and DAMPs produced by dying cells (5) (Figure 2). TLR7 recognizes single-stranded RNA (ssRNA). Its encoding gene is located on the X chromosome and frequently avoids X chromosome inactivation, which may be responsible for sex differences in type I IFN responses (12). TLR9 is an intracellular TLR expressed on endosomes and the endoplasmic reticulum that senses DNA, especially unmenthylated CpG DNA, which is more common in viruses and bacteria. After ligand-binding by TLRs, signal transduction is mediated by either the myeloid differentiation primary response 88 (MyD88)-dependent or TIR domain-containing adaptor inducing IFN-β (TRIF)-dependent pathways (11) (Figure 2). TLR3 and TLR4 signal through the TRIF-dependent pathway, and TLR4 can also signal through the MyD88-dependent pathway (11). Signaling by TLR7 and TLR9 is also MyD88 dependent. The MyD88-dependent pathway involves activation of mitogen-activated protein kinase (MAPK) and nuclear factor κ-light chain enhancer of activated B cells (NFκB), whereas TRIF-dependent signaling is mediated through phosphorylation of IRF3. Both pathways ultimately result in the induction of proinflammatory genes, including IFNs (11).

Cytosolic PRRs recognize nucleic acids, such as viral nucleic acids, but also nucleic acids arising from damaged host cells or damaged mitochondria. For instance, RIG-I and melanoma differentiation-associated gene 5 (MDA5) sense viral dsRNA in the cytosol (13, 14), relaying signals via the adaptor protein mitochondrial antiviral sensing protein (MAVS) (15). Double-stranded DNA (dsDNA) aberrantly present in the cytosol can arise from invading microbes or from the host, including through leakage of mitochondrial DNA (mtDNA) from damaged mitochondria. Cytosolic dsDNA is sensed by cGAS, which relays signals via the adaptor protein, STING (16, 17). There has been substantial recent interest in the role that cGAS-STING pathway activation plays in organ injury, and the literature describing the contributions of cGAS-STING to heart disease is reviewed in the relevant section later in this review.

### IFN signaling, IRFs and ISGs

All IFNs signal through the Janus kinase (JAK)/signal transducer and activator of transcription (STAT) pathway (Figure 1). Briefly, the intracellular domains of IFNAR1 and IFNAR2 are associated with two JAKs called non-receptor tyrosine-protein kinase 2 (TYK2) and JAK1 respectively. Ligand binding of IFNAR results in phosphorylation of the JAKs, which in turn phosphorylate tyrosine residues in the intracellular domains of the receptor subunits, as well as the STATs, STAT1 and STAT2 (18). STAT1/2 phosphorylation causes the release of the STATs from the IFNAR receptor and the formation of a trimeric complex that is comprised of STAT1, STAT2 and IRF9. This trimeric complex is called interferon-stimulated gene (ISG) factor 3, or ISGF3. ISGF3 translocates to the nucleus and acts as a transcriptional activator by binding to an interferon-stimulated response element (ISRE) in the promoter region of ISGs (5). Other type I IFN signaling pathways include the formation of STAT homodimers and heterodimers which can initiate gene transcription by binding to IFN-γ-activated site (GAS) elements in gene promoters (19). It has also been reported that IFNAR signaling can be mediated by the MAPK/c-Jun amino-terminal kinase (JNK) signaling pathway (8). Type II IFN signaling is also primarily JAK/STAT-mediated. IFN-γ binding of IFNGR1 and IFNGR2 induces association and phosphorylation of the receptor subunits with JAK1 and JAK2 respectively (5) (Figure 1). This results in STAT1 homodimerization and the binding of the STAT1 homodimers to GAS elements in gene promoter regions (5). Type III IFN signaling is similar to type I IFN signaling, involving activation of TYK2 and JAK1, recruitment of STAT1 and STAT2 and the formation of an ISGF3 complex which mediates gene transcription (20, 21) (Figure 1).

IFN responses are regulated by a group of 9 (in humans and mice) transcription factors, called IRFs. IRF proteins all contain an amino-terminal DNA binding domain that recognizes a consensus DNA sequence element called the interferon-stimulated response element (ISRE), present in the genes encoding IFNs and ISGs (5). IRFs have differing roles in regulation of IFN responses. For instance, IRF3 mediates downstream signaling relayed by the adaptor proteins TRIF, MAVS, and STING to induce the production of type I IFNs (22). In contrast, IRF2 attenuates IRF3-mediated transcriptional activation (23). The effects of IRFs, however, are not necessarily limited to their roles in the immune response. The actions of several of the individual IRFs in the heart have been described, and these actions are summarized in the relevant section later in this review.

IFNs mediate their effects by stimulating the induction of several hundred ISGs (24), which can have unique and overlapping functions. Teleologically, the end effects of IFNs and IFN genes can be considered as ways in which an infected cell can limit the damage caused to itself and to neighbouring cells by an invading microbe. For instance the ISG, protein kinase R (PKR) is a stress induced kinase that restricts protein synthesis via phosphorylation of eukaryotic initiation factor 2α (eIF2α) (25). One of the most strongly induced ISGs is the ubiquitin-like (Ubl) protein ISG15 (ISG15; also called interferon-induced 15 kDa protein). ISG15 limits the cellular damage caused by viral myocarditis (26); whereas our group recently reported induction and a pathogenetic role of ISG15 in the adverse ventricular remodeling that occurs in response to pressure overload (27). These findings illustrate the divergent effects of IFN-related pathways in the presence or absence of microbial infection, and they are elaborated upon later. In addition to its role in modulating cellular and viral protein synthesis, the IFN response also regulates the host response to viral infection by stimulating the upregulation of major histocompatibility complex class I and II antigens (28, 29), promoting programmed cell death (30–32), regulating cellular differentiation (33), suppressing angiogenesis (34), and activating other immune cells (35, 36).

### The type I IFN response and the heart

Induction of a type I IFN response has been reported to occur in both ischemic (37) and non-ischemic (27) cardiomyopathy, as well as in viral myocarditis (38). However, the contributions of the type I IFN response (being ostensibly protective or detrimental) depend on the underlying cause of the IFN response. In the context of viral infection, induction of type I IFNs has a largely protective effect (38), whereas in the absence of infection and in the setting of ischemia (37), or pressure overload (27), the type I IFN response may have deleterious consequences. Evidence of the direct cardiac effects of type I IFNs themselves can be sought through the study of genetic conditions and through clinical experience with therapeutic use of recombinant IFNs.

#### Cardiac involvement in monogenic and autoimmune interferonopathies

The monogenic type I interferonopathy Aicardi-Goutières syndrome (39) may present with an inflammatory cardiomyopathy (40); and type I IFNs have also been implicated in the pathogenesis of autoimmune congenital heart block (41). That being said, cardiac disease is not always a feature of interferonopathy. Cardiac involvement is not, for instance, a common occurrence in STING-associated vasculopathy with onset in infancy (SAVI). Likewise, whereas systemic lupus erythematosus (SLE) is associated with both an IFN gene signature (42) and an increased risk of cardiovascular disease (CVD) (43), there are insufficient data to establish a causal association between the two (44).

#### Adverse cardiac effects of recombinant IFNs

Recombinant human IFNβ1b (e.g., Betaseron) is approved for the management of multiple sclerosis. The product monograph states that there is no evidence of a direct cardiotoxic potential of Betaseron, although cases of cardiomyopathy have been reported (45). Recombinant IFNα2b (e.g., INTRON A) has regulatory approval for the management of chronic hepatitis C, chronic active hepatitis B, chronic myelogenous leukemia, multiple myeloma, non-Hodgkin's lymphoma, malignant myeloma, AIDS-related Kaposi's sarcoma, hairy cell leukemia, basal cell carcinoma, and condylomata acuminata (46). The product monograph for INTRON A states that adverse reactions associated with the cardiovascular system are mostly correlated with pre-existing CVD and prior cardiotoxic therapy, although transient reversible cardiomyopathy has been reported rarely in patients without prior evidence of cardiac disease (46). The interpretation from this experience of the use of recombinant IFNs for other indications is that the likelihood of deleterious cardiac effects of type I IFNs in otherwise normal hearts is relatively low. However, their effects in the presence of concurrent cardiac illness may be more notable. This is perhaps best exemplified by a report describing the role of IRF3 and type I IFNs in the response to myocardial infarction (MI) (37).

#### IRF3 and the type I IFN response to MI

In a landmark study published in 2017, King and co-workers used single cell RNA sequencing (scRNA-seq) to profile leukocytes isolated from the hearts of mice after MI (37). In doing so, they observed that a subtype of cardiac macrophages was characterized by IRF3/type I IFN activation, and that disruption of either IRF3 or IFNAR signaling resulted in improved survival following MI, decreased inflammation and improved cardiac function (37). The authors concluded that the high level of cell death that occurs in MI results in disruption of the compartmentalization of DNA in the cell nucleus and mitochondria and interferes with the housekeeping actions of self-DNases. This leads to the release of damage signals, especially dsDNA from dying cells (37). dsDNA, in turn, is sensed by cGAS in infiltrating phagocytes leading to activation of an IRF3-dependent type I IFN response (37). Secreted IFNs then diffuse in the local microenvironment, binding to IFNARs on neighbouring cells and amplifying the type I IFN response through induction of ISGs (37). In our opinion, this postulated mechanism most clearly exemplifies how type I IFNs may be induced during myocardial injury and how the type I IFN response may, in turn, contribute to adverse cardiac outcomes.

#### ISG15 in viral myocarditis and pressure overload

**Figure 3:**
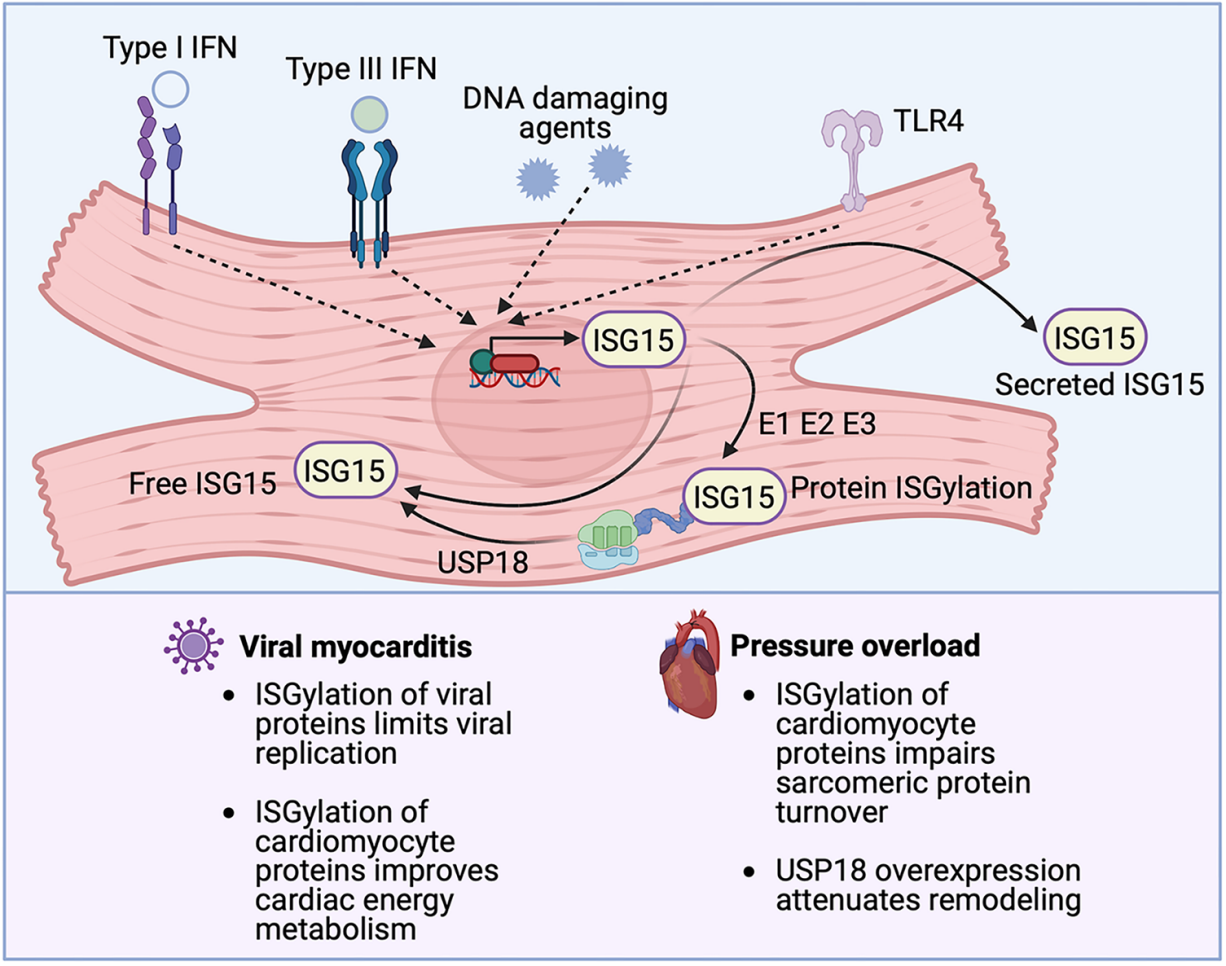
The emerging role of ISG15 in heart disease. ISG15 is one of the most strongly inducible interferon (IFN) stimulated genes, the expression of which can be triggered by IFNs themselves, DNA damaging agents or TLR signaling. ISG15 exists in 3 forms, an intracellular free form, a secreted form and a protein-bond form. ISG15 binds to newly synthesized viral or host proteins through an energy consuming process, termed ISGylation, that requires E1-activating, E2-conjugating, and E3-ligating enzymes. Removal of ISG15 from proteins (de-ISGylation) is mediated by the protease USP18. ISG15 induction protects against viral myocarditis, likely through the ISGylation of both viral and cardiomyocyte proteins. Conversely, in the absence of microbial infection, ISG15 induction can have detrimental effects, as has been observed in pressure overload, where the ISGylation of newly synthesized cardiomyocyte proteins impairs sarcomeric protein turnover.

Whereas the example above illustrates the potentially deleterious effects of the type I IFN response in MI, type I IFNs are protective against viral myocarditis. For instance, mice deficient in IFNAR are susceptible to coxsackievirus B3 (CVB3) infection (47), and mice deficient in IFN-β experience exacerbated CVB3-induced myocarditis (48). The example of ISG15 illustrates how a downstream effector ISG of the type I IFN response also may play a context-dependent role in the protection from or development of cardiac injury. ISG15 is a Ubl protein and, in this role, once it is induced ISG15 can post-translationally modify lysine residues on actively translated proteins, including viral proteins (49) (Figure 3). The post-translational modification of proteins by conjugation with ISG15 is termed, ISGylation. In addition to its intracellular effects, ISG15 also exists in an intracellular free form and it can also be secreted. In its secreted form, ISG15 binds to its receptor, lymphocyte function-associated antigen 1 (LFA-1) expressed especially by T cells and NK cells, where it stimulates IFN-γ production (50, 51) (Figure 3). Absence of ISG15 has been reported to exacerbate CVB3 myocarditis (26), and this effect was also mimicked in mice lacking ubiquitin-activating enzyme E1-like (UBE1l), the E1-activating enzyme necessary for protein ISGylation (26). The authors of that article attributed the protective actions of ISG15 to the ISGylation of CVB3 2A protease, limiting CVB3-induced cleavage of host eukaryotic initiation factor 4γ (eIF4G) in cardiomyocytes, which ordinarily promotes viral infection by restricting host cell protein translation (26). A recent study, further illuminated the role of ISG15 in viral myocarditis, concluding that induction of ISG15 in myocarditis functions to counter cardiac atrophy and dysfunction by increasing the heart's metabolic capacity through downregulation of cardiac glycolysis and enhancing the respiratory activity of mitochondria (52).

Viral infection, however, is not the only cause of cardiomyocyte ISG15 induction. For instance, Maier and coworkers reported that cardiomyocytes with constitutively active IκB kinase/NFκB signaling exhibited a type I IFN response that is characterized by activation of the ISG15 pathway (53). In the absence of viral infection, the conjugation of ISG15 to actively translated host proteins can affect several cellular processes including cytoskeletal dynamics, DNA damage responses, cytokine release, and immune modulation (54). In recent work by our group, we set out to determine the mechanism(s) by which proinflammatory CCR2-expressing macrophages contribute to pressure overload-induced ventricular remodeling (27). We found that exposure of cardiomyocytes to the secreted products of CCR2+ cardiac macrophages isolated from mouse hearts, induced a profound type I IFN response, characterized by ISG15 induction. Cardiac ISG15 induction was also observed in mouse hearts after transverse aortic constriction (TAC) or following infusion with angiotensin II, the left ventricles (LVs) of uninephrectomized rats treated with deoxycorticosterone acetate (DOCA) salt, and the right ventricles of rats after pulmonary artery banding (27). We observed that, in pressure overload, ISG15 induction results in the ISGylation of newly translated cardiomyocyte proteins, including the myofibrillar protein, filamin-C (27), and that absence of ISG15 attenuated adverse ventricular remodeling after TAC (27). Interestingly, in that study, we also found cardiac ISG15 levels to be markedly reduced in *Ifnar1* deficient mice even in the absence of pressure overload (27). This observation illustrates that constitutively expressed IFNs can have important physiological roles even in the absence of induction and even though they are present at very low levels (55).

Protein ISGylation is mediated by an energy-consuming process involving E1-activating enzymes, E2-conjugating enzymes, and E3-ligating enzymes and it is reversed by an ISG15-specific protease called ubiquitin-specific protease 18 (USP18), which itself is an ISG (56–58) (Figure 3). Whereas we reported a pathogenetic role for protein ISGylation in pathological ventricular hypertrophy, Ying and coworkers described a protective effect of USP18 overexpression, which would be expected to reverse protein ISGylation (59). In brief, the authors observed that cardiomyocyte-specific overexpression of USP18 attenuated myocardial hypertrophy, fibrosis, ventricular dilatation, and ejection fraction decline induced by aortic banding, whereas USP18 knockout exacerbated remodeling (59). The authors attributed the cardioprotective effects of USP18 to inhibition of transforming growth factor β-activated kinase 1 (TAK1)/MAPK/JNK signaling (59). They had focused on this pathway because USP18 had previously been reported to deubiquitinate TAK1 (60), whereas the polyubiquitination of TAK1 is important for its autoactivation and downstream activation of p38 MAPK/JNK signaling (61). Other studies, though, have reported that USP18 is specific for ISG15 and that USP18 does not remove ubiquitin from substrate proteins (62, 63). Accordingly, it is feasible that the phenotypes observed in the USP18 overexpressing/knockout mice were mediated through altered protein ISGylation, although this possibility was not explored in the original report (59).

Lastly, whereas the role of ISG15 in the defence against viral infection has been known about for decades (64), its biological function in this capacity has gained increasing attention of late because of the involvement of ISG15 in COVID-19, with potentially intracellular and extracellular proviral and antiviral effects (65). The contribution of an insufficient or augmented IFN response to COVID-19 severity and its potential cardiac complications is discussed later in this review.

### The emerging role of cGAS-STING pathway activation in the pathogenesis of heart disease

**Figure 4:**
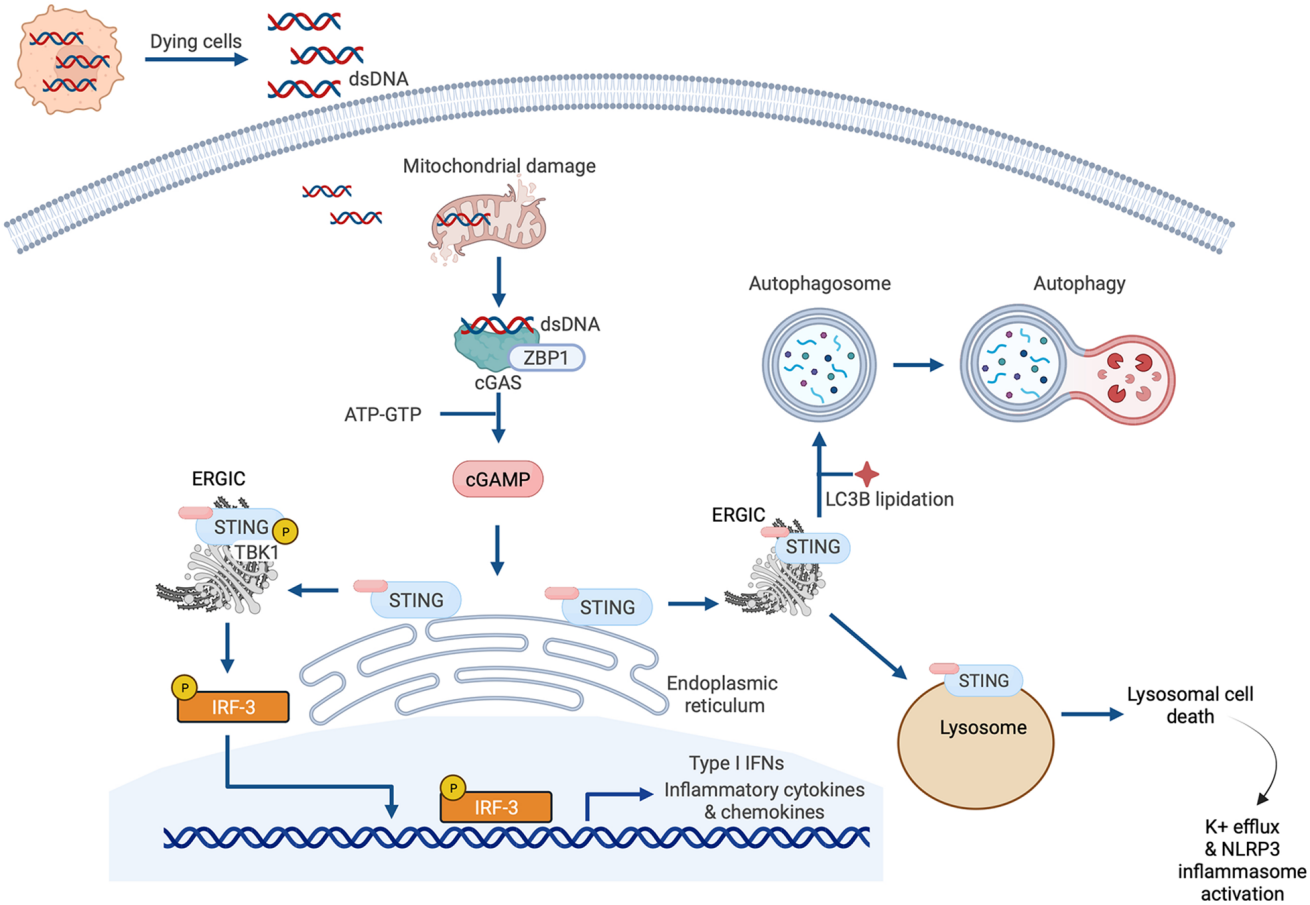
Cyclic GMP-AMP synthase (cGAS)-stimulator of interferon genes (STING) signaling. The canonical cGAS/STING pathway is initiated by the aberrant presence of double-stranded DNA (dsDNA) in the cytosol, which can originate from invading microbes but also from dying cells and damaged mitochondria. dsDNA binds to cGAS and induces the synthesis of cGAMP from ATP and GTP, which in turn induces STING activation and the translocation of STING to the endoplasmic reticulum-Golgi intermediate compartment (ERGIC) and the Golgi. There, STING recruits and activates TANK-binding kinase 1 (TBK1), which phosphorylates STING and recruits IRF3 to the TBK1-STING complex. TBK1 phosphorylates IRF3, causing IRF3 dimerization, nuclear translocation, and target gene induction. STING can also induce nuclear factor κ-light chain enhancer of activated B cells (NFκB) activation. STING has also recently been reported to be a proton channel and, in this role, it acts as a source for LC3B lipidation which is important for autophagy; and triggers lysosomal cell death and NLRP3 inflammasome activation.

The earlier summarized study by King et al. that reported the importance of IRF3 and the type I IFN response in MI described a central role for cGAS-STING signaling in myocardial injury (37). This is one of several recent studies espousing the significance of cGAS-STING pathway activation in cardiac disease that have emerged since the original description of the pathway in 2013 (66, 67). Briefly, the cGAS-STING signaling pathway exists to mediate the immune response to displaced dsDNA which can originate from invading microorganisms, dead cells, extracellular vesicles, or leakage of DNA from damaged mitochondria (Figure 4). dsDNA binds to cGAS and activates the synthesis of the second messenger 2′3′ cyclic GMP-AMP (cGAMP) from ATP and GTP. cGAMP, in turn, binds to the active pocket site of the dimeric adaptor protein STING, causing STING activation and downstream signaling (68). Once it is activated, STING translocates from its residing place in the endoplasmic reticulum (ER) to the endoplasmic reticulum-Golgi intermediate compartment (ERGIC) and the Golgi where it recruits and activates TANK-binding kinase 1 (TBK1), which in turn phosphorylates STING [on serine residue 366 (Ser366) in humans, Ser365 in mice], and recruits IRF3 to the TBK1-STING complex (22, 69) (Figure 4). TBK1 phosphorylates IRF3, causing IRF3 dimerization, nuclear translocation and induction of its target genes (69), including type I IFNs, ISGs and inflammatory cytokines (70) (Figure 4). In addition to this canonical mechanism of gene induction by cGAS-STING, STING can also induce NFκB activation (68). The role of TBK1 in NFκB activation is unclear. It has been suggested that TBK1 is dispensable for STING activation of NFκB, and that this process requires TAK1 and IκB kinase (IKK) complexes in myeloid cells (71). However, other authors have reported that TBK1 recruitment is necessary for STING-mediated NFκB activation (72). Aside from (and independent of) its role in mediating the induction of IFNs and cytokines, STING also plays an important role in autophagy induction (73), and inflammasome activation (74). For instance, when STING binds cGAMP and translocates to the ERGIC, the STING-containing ERGIC acts as a source for non-canonical LC3B lipidation which is important for the biogenesis of autophagosomes (73) (Figure 4). STING-induced autophagosome formation is dependent on a direct interaction between STING and WD repeat domain, phosphoinositide interacting 2 (WIPI2) (75). Separately, activated STING traffics to the lysosome where it triggers membrane permeabilization and lysosomal cell death, and potassium efflux which promotes NLRP3 inflammasome activation (74) (Figure 4). Interestingly, human STING has also recently been reported to act as a proton channel, and its effects in both L3B lipidation and inflammasome activation have been attributed to this property (76).

Experimental studies implicating cGAS-STING pathway activation in the pathogenesis of heart disease are summarized in Table 1. Some of the findings from these studies are elaborated upon below.

**Table 1:** Experimental studies implicating the cGAS-STING pathway in heart disease.

Years	Disease or experimental context	Summary of principal findings	Citation
2017	Myocardial infarction	Mice with functionally deficient STING mimic IRF3 knockout mice in their gene expression patterns after MI	(37)
2018	Myocardial infarction	cGAS knockout improved survival in mice after MI, decreased pathological remodeling, enhanced angiogenesis, and preserved contractile function	(77)
2020	Pressure overload	AAV9-mediated knockdown of cGAS with shRNA improved survival, preserved LV contractility, and attenuated pathological remodeling in mice after TAC	(78)
2020	High fat diet	High fat diet upregulated cGAS-STING and augmented cardiac remodeling in Akt2-AMPK double knockout mice, and cGAS-STING inhibition attenuated cardiomyocyte contractile dysfunction	(79)
2020	Alzheimer's disease	Knockdown of cGAS or STING negated the beneficial effects of melatonin on neonatal cardiomyocyte mitophagy and apoptosis in response to APP/PS1 mutation	(80)
2020	Cigarette smoke	cGAS-STING inhibition rescued smoke-induced contractile dysfunction in wildtype and *Beclin1* haploinsufficient (*Becn*^+/−^) neonatal cardiomyocytes, except cGAS inhibition in *Becn*^+/−^ cardiomyocytes	(81)
2022	Reperfused myocardial infarction	Small molecule STING inhibition decreased infarct expansion, attenuated cardiac function decline, and reduced myocardial hypertrophy	(82)
2022	Myocardial infarction	Small molecule STING inhibition preserved cardiac function and attenuated fibrosis in mice with MI	(83)
2022	Diabetic cardiomyopathy	AAV9-mediated knockdown of STING with shRNA preserved cardiac function and hypertrophy and alleviated cardiac pyroptosis in streptozotocin-diabetic high fat diet-fed mice	(84)
2023	Doxorubicin-induced cardiotoxicity	AAV9-mediated knockdown of STING with shRNA improved survival and cardiac function and reduced inflammation in mice with doxorubicin cardiotoxicity	(85)
2023	Cardiac transplantation	Graft survival was prolonged in donor hearts from cGAS knockout mice	(86)
2023	Doxorubicin-induced cardiotoxicity	Global deficiency of cGAS, STING or IRF3 each ameliorated doxorubicin-induced cardiotoxicity in mice; and endothelial-specific STING deficiency attenuated cardiotoxicity and endothelial dysfunction	(87)
2023	Doxorubicin-induced cardiotoxicity	ZBP1 stabilizes Z-form mtDNA and cooperates with cGAS to induce type I IFN signaling and cardiotoxicity induced by doxorubicin	(88)
2023	Cholesterol metabolism	Carnitine acetyltransferase depletion induced mtDNA stress and a cardiomyocyte innate immune response mediated by cGAS-STING	(89)

cGAS, cyclic GMP-AMP synthase; STING, stimulator of interferon genes; MI, myocardial infarction; IRF, interferon regulatory factor; AAV9, adeno-associated virus 9; shRNA, short hairpin RNA; LV, left ventricle; TAC, transverse aortic constriction; AMPK, AMP-activated protein kinase; APP, amyloid precursor protein; PS1, presenilin 1; ZBP1, Z-DNA binding protein 1; mtDNA, mitochondrial DNA.

#### cGAS-STING in MI

In 2018 Cao and coworkers reported that MI caused by ligation of the left anterior descending (LAD) artery induced upregulation of the cGAS-STING pathway, which sustains the inflammatory “M1-like” macrophage phenotype (77). Furthermore, inactivation of the pathway through knockout of cGAS prompted a more “M2-like” macrophage phenotype that was accompanied by improved wound healing, enhanced angiogenesis, diminished remodeling, and improved survival (77). Similarly, treatment of mice with the STING antagonist H-151 has been reported to improve outcomes after experimental MI (82, 83).

#### cGAS-STING in non-ischemic cardiomyopathy

Zhang and coworkers reported STING upregulation in heart tissue of humans with dilated cardiomyopathy or HCM, and in the hearts of mice with pressure overload induced by aortic banding (90). In that study, knockout of STING attenuated pathological hypertrophy and ejection fraction decline induced by aortic banding (90). Similarly, Hu et al. also observed activation of the cGAS-STING pathway in mice with ventricular remodeling caused by TAC, with a preservation of LV function (and improved survival) when cGAS was knocked down using adeno-associated virus 9 (AAV9) gene transfer of short hairpin RNA (shRNA) (78).

#### cGAS-STING in diabetes and in sepsis

Yan et al. reported that oxidative damage-induced mtDNA leak was accompanied by cGAS-STING pathway activation in the hearts of diabetic high fat diet-fed mice, and that AAV9-mediated knockdown of STING with shRNA preserved cardiac function (84). In a model of sepsis-induced cardiac injury, Li et al. observed that global STING knockout attenuated LV systolic dysfunction and improved survival in mice injected with LPS (91).

#### Noncanonical actions of cGAS-STING in the heart

The above examples, and those additional studies summarized in Table 1, illustrate how a substantial body of literature has arisen in recent years indicating that cGAS-STING pathway activation takes place in both ischemic and non-ischemic cardiomyopathy and that blockade of cGAS-STING signaling improves cardiac outcomes in experimental models. More recently still, new fundamental insights into cGAS-STING biology have emerged through the study of the actions of the pathway in the heart. For example, one recent study reported that cGAS interacts with the innate immune sensor protein Z-DNA binding protein 1 (ZBP1) to promote type I IFN responses and cardiotoxicity (88). Briefly, because mtDNA is circular and lacks free ends it cannot rotate to relieve torsional stress. As a result, mitochondrial genome instability promotes the accumulation of a form of DNA called Z-DNA which differs from classical Watson-Crick B-DNA in its conformation including (but not limited to) that Z-DNA is a left-handed double helix, and B-DNA is a right-handed double helix. Mitochondrial Z-DNA is stabilized by ZBP1 which nucleates a complex that contains cGAS, as well as the mediators of cell death and inflammation, receptor-interacting protein 1 (RIPK1) and RIPK3 (88). This, in turn, augments STAT1 phosphorylation, and induces type I IFN signaling that is dependent on ZBP1, STING and IFNAR (88). Illustrating the importance of the interaction between ZBP1 and cGAS in mtDNA-sensing and downstream signaling, mice lacking ZBP1, STING or IFNAR1 were protected from doxorubicin-induced cardiotoxicity (88). Interestingly though, ZBP1 is not required for sensing of cytosolic B-form mitochondrial DNA (88). Separately, in another recent study, Mao et al. implicated cGAS-STING activation as an important mediator of the mechanisms by which altered cholesterol metabolism may affect innate immune responses in the heart (89). In that study, the investigators reported that depletion of carnitine acetyltransferase (CRAT) from cardiomyocytes promoted cholesterol catabolism, and accumulation of bile acid and the intermediate 7α-hydroxyl-3-oxo-4-cholestenoic acid (89). This, in turn, induced mitochondrial stress and cGAS-STING-dependent type I IFN responses, which contributed to myocardial inflammation and heart failure (89).

### IFN-γ

**Figure 5:**
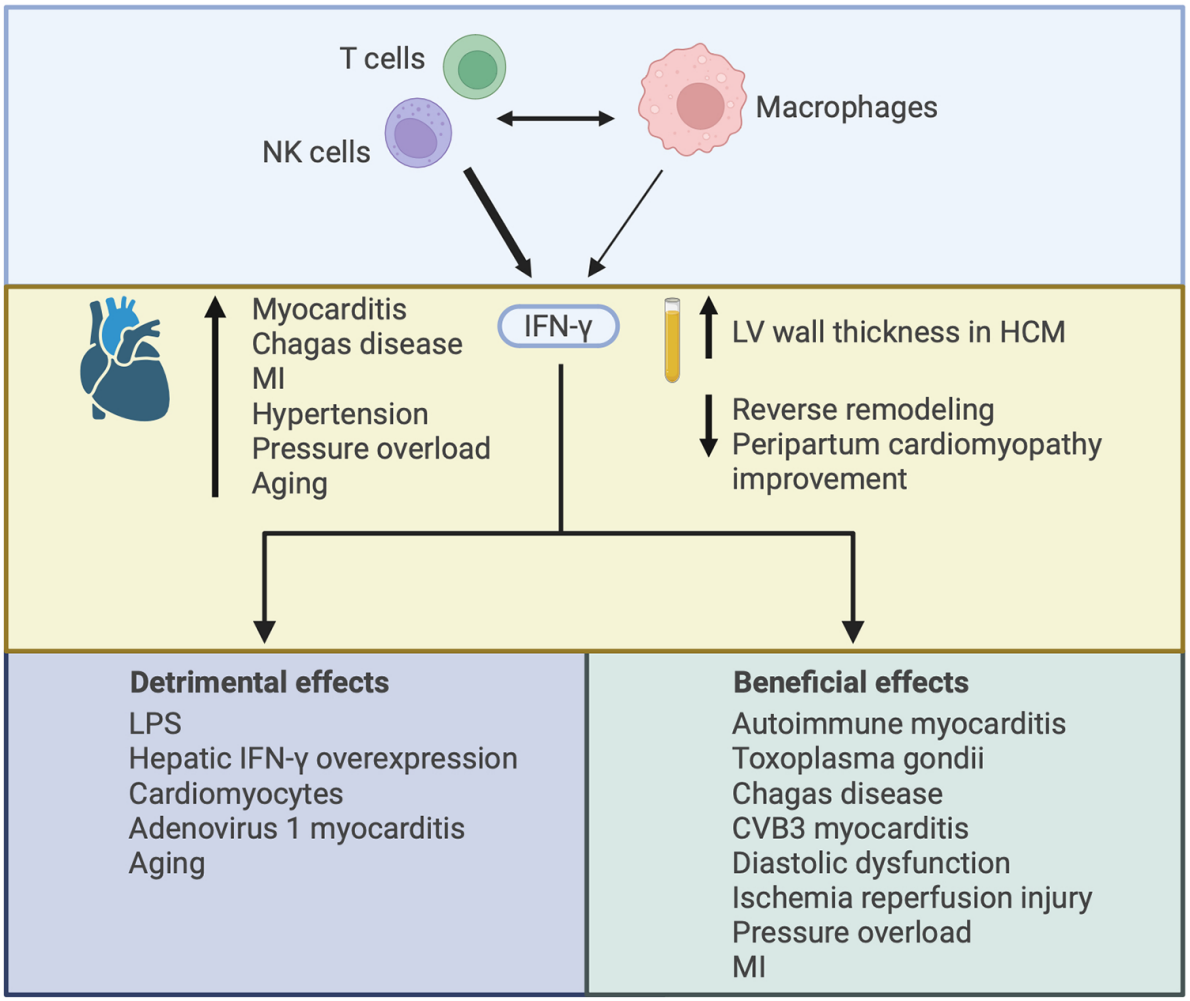
Illustrating the varied effects of interferon-γ (IFN-γ) in the heart. IFN-γ is mainly produced by CD4+ and CD8+ T cells and NK cells, with some contribution from macrophages. Interaction between adaptive and innate immune cells can drive IFN-γ production by each cell-type. IFN-γ expression in the heart has been described in several disease settings and increased plasma levels of IFN-γ have been associated with worsened cardiac outcomes. The increase in IFN-γ may contribute to cardiac injury or it may be compensatory, with both detrimental and beneficial effects of IFN-γ described according to the nature of IFN-γ augmentation or deficiency and the context in which it has been studied. HCM, hypertrophic cardiomyopathy; MI, myocardial infarction; LPS, lipopolysaccharide.

IFN-γ in the heart arises from infiltrating inflammatory cells, primarily CD4+ and CD8+ T cells and NK cells (92, 93), with some contribution from macrophages (92, 94) (Figure 5). Interaction between T cells and macrophages is important in driving IFN-γ production by each cell-type, and IFN-γ plays a central role in mediating crosstalk between innate and adaptive immune cell populations (92, 95). Cardiac IFN-γ levels are increased in a range of different diseases including myocarditis (96), Chagas disease cardiomyopathy (97–99), MI (100), hypertensive heart disease (101), pressure overload (94), and aging (102) (Figure 5). In comparison to the type I IFNs, a much larger body of literature exists attesting to the actions of IFN-γ in the heart, with overall conflicting reports describing both beneficial and detrimental context-dependent effects of IFN-γ. These studies are summarized in Table 2 and Figure 5, and some of the key observations are discussed below.

**Table 2:** Experimental studies reporting the detrimental or beneficial effects of IFN-γ in the heart.

Years	Disease or experimental context	Reported actions of IFN-γ	Citation
Detrimental effects of IFN-γ			
1998	Rat papillary muscle	Cardiodepressant effect of IFN-γ in the presence of LPS	(103)
2001	Isolated retrograde-perfused rat hearts	Augmented depression of inotropic and lusitropic effects of LPS and IFN-γ in aged hearts	(104)
2007	Hepatic IFN-γ overexpression	Chronic active myocarditis and cardiomyopathy in IFN-γ overexpressing mice	(105)
2012	Hepatic IFN-γ overexpression	TNFα knockout attenuated myocarditis and cardiomyopathy in IFN-γ overexpressing mice	(106)
2012	Neonatal rat ventricular myocytes	Cardiac myocyte atrophy induced by recombinant IFN-γ due to degradation of myosin heavy chain protein	(107)
2015	Mouse adenovirus 1 (MAV-1)	Attenuated myocarditis with neutralizing antibody mediated IFN-γ depletion	(96)
2021	AC16 cardiomyocytes	Co-incubation with IFN-γ and TNFα induced cardiomyocyte mitochondrial dysfunction and nitro-oxidative stress	(99)
2023	Aging	IFN-γ response signature in aging mouse hearts mimicked by recombinant IFN-γ treatment of induced pluripotent stem cell derived cardiomyocytes, accompanied by reductions in oxidative phosphorylation and glycolysis	(102)
Beneficial effects of IFN-γ			
2001	Autoimmune myocarditis	Exacerbated cardiac α-myosin heavy chain induced autoimmune myocarditis in IFN-γ receptor knockout mice	(108)
2001	Toxoplasma gondii	Augmented accumulation of Toxoplasma gondii in the hearts of IFN-γ knockout mice	(109)
2001	Chagas disease	Augmented cardiac Trypanosoma cruzi parasitism in IFN-γ knockout mice	(110)
2004	Autoimmune myocarditis	Constrictive pericarditis and augmented myocarditis in IFN-γ knockout mice injected with cardiac myosin	(111)
2004	Viral myocarditis	Worsened CVB3-induced myocarditis in IFN-γ knockout mice	(112)
2005	PGF(2α)-treated rat cardiac myocytes and abdominal aortic constriction	Recombinant IFN-γ attenuated myocardial hypertrophy	(113)
2012	Aldosterone infusion, uninephrectomy and 1% saline water	Augmented LV hypertrophy and worsened diastolic dysfunction in IFN-γ knockout mice	(114)
2013	Porcine cardiopulmonary bypass-associated myocardial ischemia-reperfusion injury	Preconditioning with IFN-γ improved recovery of ventricular function after ischemia reperfusion injury	(115)
2018	Pressure overload	Worsened hypertrophy, cardiac fibrosis and dysfunction in IFN-γ knockout mice after TAC	(94)
2019	Left anterior descending artery ligation	IFN-γ orchestrated the sequential cellular immune response after MI and IFN-γ knockout impaired cardiac function and survival after MI	(100)

Table does not include studies reporting solely an association of IFN-γ with cardiac or atherosclerotic disease, or the role of IFN-γ in the immune response to transplantation.

IFN-γ, interferon-γ; LPS, lipopolysaccharide; TNFα, tumor necrosis factor α; MAV-1, mouse adenovirus 1; CVB3, coxsackievirus B3; PGF(2α), prostaglandin F2α; LV, left ventricle, TAC, transverse aortic constriction; MI, myocardial infarction.

#### Studies reporting deleterious actions of IFN-γ in the heart

Transgenic mice that overexpress IFN-γ in their livers, and thus with high circulating serum levels of IFN-γ, have been reported to develop a chronic active myocarditis and cardiomyopathy characterized by accumulation of CD4+ and CD8+ T cells, macrophages and dendritic cells (105). This was accompanied by upregulation in the expression of proinflammatory cytokines including TNFα, IL-12, CCL2 and CCL3 (105), illustrating the role of IFN-γ in inflammatory gene induction. Furthermore, in patients with LV assist device (LVAD) implantation, low serum IFN-γ (and TNFα) predicted cardiac improvement (116). This is interesting because, in experimental studies, co-exposure of cardiomyocytes to IFN-γ and TNFα has also been reported to induce mitochondrial dysfunction and nitro-oxidative stress (99). These findings suggesting deleterious actions of IFN-γ in the heart are supported by several other studies in experimental animals (92), isolated atria (117), cultured cardiomyocytes (99), and cultured cardiac fibroblasts (118), reviewed in (95). Given the production of IFN-γ by infiltrating adaptive immune cells, and the role of IFN-γ in orchestrating the interaction between innate and adaptive immune cells, this seems teleologically appropriate. However, several other studies have described a protective role for IFN-γ in the heart.

#### Studies reporting protective actions of IFN-γ in the heart

IFN-γ knockout mice have been reported to develop worsened hypertrophy, cardiac fibrosis and cardiac dysfunction after TAC (94). This has been attributed to an essential role of IFN-γ in Stat5-dependent activation of phosphoinositide 3-kinase/Akt signaling during compensatory hypertrophy (94). Cardiac inflammation has also been reported to be increased in IFN-γ knockout mice injected with cardiac myosin and accompanied by a constrictive pericarditis (111). Furthermore, cardiac hypertrophy and diastolic dysfunction were exacerbated in IFN-γ knockout mice with aldosterone, uninephrectomy and salt water feeding (114), whereas recombinant IFN-γ attenuated cardiac hypertrophy in rats with abdominal aorta banding (113). Other reports also describing beneficial actions of IFN-γ in the heart are also summarized in Table 2.

In sum, cardiac IFN-γ is increased in several different disease states. Its role in these diseases may contribute to their pathogenesis, or it may be compensatory, with experimental studies reporting both deleterious and protective actions of IFN-γ. These actions appear to be dependent on the models studied, timing of intervention, mechanism of upregulation or inhibition, and the experimental endpoints employed.

### Interferon regulatory factors

The IRFs are a family of 9 transcription factors that play an important role in the immune response, also regulating other cellular processes. IRF1 regulates gene expression by binding to ISREs in their promoter regions (119) and, although it is best studied as a transcriptional activator (119), IRF1 can also function as a transcriptional repressor (120). IRF2 competitively inhibits IRF1-mediated gene transcription (121). IRF3, IRF5 and IRF7 are important for the production of type I IFNs in response to PRR-mediated signaling (122). IRF9 regulates IFN-induced gene expression, and IRF4, IRF5 and IRF8 control myeloid cell development and responses (122). IRF6 is important for early development (123). Most of the IRFs have been studied individually for their role in the heart, and these actions are summarized below and in Table 3.

**Table 3:** Experimental studies exploring the actions of interferon regulatory factors (IRFs) in cardiac disease.

IRF	Years	Disease or experimental context	Reported actions of IRFs	Citation
IRF1	2014	Pressure overload	Cardiac IRF1 overexpression exacerbated hypertrophy, ventricular dilatation and dysfunction, whereas IRF1 knockout attenuated cardiac hypertrophy	(124)
IRF1	2020	Cardiorenal syndrome type 4	IRF1 mediated cardiac PGC1α downregulation and consequent dysfunction of myocardial energy metabolism	(125)
IRF2	2021	Myocardial infarction	IRF2 contributed to cardiac dysfunction in MI by inducing gasdermin D-mediated pyroptosis	(126)
IRF3	2011	Angiotensin II induced cardiac fibrosis	IRF3 knockout attenuated cardiac fibrosis	(127)
IRF3	2013	Aortic banding	IRF3 knockout exacerbated cardiac hypertrophy, whereas cardiac IRF3 overexpression attenuated it	(128)
IRF3	2017	Myocardial infarction	MI induced activation of an IRF3-type I IFN axis in cardiac macrophages which was caused by release of DNA from damaged cells, and which impaired cardiac function	(37)
IRF4	2013	Aortic banding	Cardiac specific overexpression of IRF4 exacerbated hypertrophy, fibrosis, and dysfunction and IRF4 knockout attenuated cardiac hypertrophy	(129)
IRF5	2014	Myocardial infarction	Nanoparticle-delivered siRNA against IRF5 supported infarct healing and attenuated heart failure after MI	(130)
IRF5	2019	Viral myocarditis	IRF5 interference attenuated viral myocarditis	(131)
IRF7	2014	Aortic banding	Cardiac specific overexpression of IRF7 attenuated hypertrophy, fibrosis, and dysfunction and IRF7 knockout augmented cardiac hypertrophy and fibrosis	(132)
IRF7	2022	Cardiac autoinflammation	IRF7 knockout attenuated cardiac autoinflammation induced by ADAR1 inactivation	(133)
IRF8	2014	Aortic banding	Cardiac specific overexpression of IRF8 attenuated hypertrophy and fibrosis, and IRF8 knockout augmented cardiac hypertrophy and fibrosis	(134)
IRF9	2013	Aortic banding	Cardiac-specific overexpression of IRF9 attenuated cardiac hypertrophy whereas hypertrophy, fibrosis and cardiac dysfunction were augmented in IRF9 knockout mice	(135)

Table does not include studies reporting solely an association of IRFs with cardiac or atherosclerotic disease, or the role of IRFs in the immune response to transplantation. No experimental studies were identified that examined the role of IRF6 in cardiac disease.

IRF, interferon regulatory factor; PGC1α, peroxisome proliferator-activated receptor gamma activator 1-alpha; MI, myocardial infarction; siRNA, short interfering RNA; ADAR1, adenosine deaminase acting on RNA-1.

Several of these studies were published by the same team in 2013–2014, and followed a similar pattern of investigation involving: exploration of change in IRF protein levels in diseased hearts; cardiac-specific IRF overexpression and global IRF knockout; and elucidation of a cellular mechanism underlying the beneficial or detrimental cardiac effects of IRF overexpression and knockout (124, 128, 129, 132, 134, 135).

#### IRF1

IRF1 expression has been reported to be reduced in the hearts of humans with dilated cardiomyopathy or hypertrophic cardiomyopathy, whereas in mice with pressure overload caused by banding of the thoracic aorta there was an early upregulation of IRF1 after 3–7 days, followed by a reduction in IRF1 protein in the heart by weeks 4–6 (124). Cardiac-specific IRF1 overexpression exacerbated pressure overload-induced hypertrophy, ventricular dilatation and dysfunction, whereas cardiac hypertrophy was attenuated in IRF1 knockout mice and rats (124). These prohypertrophic effects of IRF1 were attributed to induction of inducible nitric oxide synthase (iNOS) caused by binding of IRF1 to the promoter region of the iNOS gene (124). IRF1 has also been implicated in the pathogenesis of cardiac dysfunction that can occur because of chronic kidney disease (CKD), termed cardiorenal syndrome type 4 (125). In that study, the authors reported that high phosphate levels in CKD impair myocardial energy metabolism by downregulating the transcription of the master regulator of mitochondrial biogenesis, peroxisome proliferator-activated receptor gamma activator 1-alpha (PGC1α) (125). Mechanistically, high phosphate was observed to epigenetically regulate the expression of IRF1 by inducing acetylation of histone protein H3 on lysine residue 9 (H3K9) (125). IRF1 has a repressor domain and, in some circumstances, can bind to the IRF response element of target genes to repress gene expression (136, 137). In the case of cardiorenal syndrome type 4, IRF1, induced by high phosphate, was observed to bind directly to the promoter region of the PGC1α encoding gene inhibiting PGC1α transcription (125).

#### IRF2

IRF2 levels have been reported to be increased after experimental MI, whereas lentiviral mediated silencing of IRF2 with shRNA attenuated cardiac dysfunction after MI, an effect attributed to the role of IRF2 in gasdermin D-mediated pyroptosis (126).

#### IRF3

Several studies have explored the effects of IRF3 in the heart. In 2011, Tsushima and coworkers reported that IRF3 knockout attenuated cardiac fibrosis and ventricular chamber shrinkage in mice infused with angiotensin II, whereas cardiac hypertrophy was unaffected (127). In that study, the authors attributed IRF3 activation by angiotensin II in cardiac fibroblasts to be mediated by ERK signaling rather than canonical TBK1/IKK signaling (127). Lu and coworkers performed aortic banding in IRF3 knockout mice and in mice with cardiac-specific IRF3 overexpression, observing that IRF3 knockout exacerbated cardiac hypertrophy and IRF3 overexpression attenuated it, and concluding that IRF3 is a negative regulator of pathological cardiac hypertrophy (128). They attributed this effect to an interaction between IRF3 and ERK2, which inhibited ERK1/2 signaling (128). The authors also observed that IRF3 levels are increased early after aortic banding and return to basal levels by day 28, whereas IRF3 is also upregulated in human failing hearts (128). Considering the more recent findings of King et al, using scRNA-seq (37), it seems likely that the early increases in cardiac IRF3 levels in mice after aortic banding and in human heart failure may represent accumulation of cardiac macrophages, which is known to occur early after aortic banding (138).

#### IRF4

Jiang et al. reported that IRF4 is downregulated in human dilated cardiomyopathy, in mouse hearts 4 and 8 weeks after aortic banding, and in cardiomyocytes exposed to angiotensin II or phenylephrine (129). They found that cardiac specific overexpression of IRF4 exacerbated pressure overload-induced hypertrophy, fibrosis, and dysfunction, whereas IRF4 knockout attenuated cardiac hypertrophy (129). The authors attributed this effect to a role for IRF4 in inducing the transcription of cAMP response element-binding protein (CREB) in cardiomyocytes, which is known to promote cardiac hypertrophy (139, 140).

#### IRF5

IRF5 plays a key role in macrophage polarization favoring an “M1-like” phenotype (141, 142). In 2014, Courties et al. used nanoparticle-delivered siRNA to silence IRF5 in infarct macrophages and observed that IRF5 knockdown attenuated the development of heart failure after MI in ApoE knockout mice (130). Whereas most IFNs and IFN-related pathways have shown to have protective roles in viral myocarditis, IRF5 may play a role in the pathogenesis of cardiac injury in this setting. Specifically, Nie et al. observed upregulation of the TLR9-IRF5 pathway in the hearts of humans and mice with CVB3 myocarditis, and they observed that an AAAG-rich oligodeoxynucleotide that interferes with IRF5 alleviated myocarditis in CVB3-infected mice (131).

#### IRF7

Jiang et al. also studied the actions of IRF7, observing that IRF7 negatively regulates cardiac hypertrophy (132). Briefly, the authors reported that either angiotensin II or phenylephrine decreased IRF7 protein levels in neonatal rat cardiomyocytes, and IRF7 protein levels were also observed to be reduced in the hearts of mice 2 and 4 weeks after aortic banding (132). Cardiac specific IRF7 overexpression attenuated pressure overload-induced hypertrophy, fibrosis, and dysfunction, whereas IRF7 knockout augmented cardiac hypertrophy and fibrosis (132). These effects were attributed by the authors to binding of IRF7 to inhibitor of κB kinase-β (IKKβ) and consequent inactivation of NFκB (132). Elsewhere, IRF7 expression levels have been reported to be markedly increased in the hearts of mice with CVB3 myocarditis (143). More recently, inactivation of adenosine deaminase acting on RNA-1 (ADAR1) (which acts as an RNA sensing inhibitor) was found to induce a late-onset autoinflammatory myocarditis, dilated cardiomyopathy, and heart failure (133). This phenotype was attenuated by IRF7 knockout, indicating that IRF7 is the principal mediator of cardiac autoinflammation induced by ADAR1 absence (133).

#### IRF8

When studying the cardiac effects of IRF8, Jiang and coworkers observed a reduction in cardiac IRF8 protein levels in the hearts of humans with dilated cardiomyopathy or hypertrophic cardiomyopathy, mice with pressure overload caused by aortic banding, and in neonatal rat cardiomyocytes incubated with angiotensin II or phenylephrine (134). Mice with cardiac-specific overexpression of IRF8 were resistant to hypertrophy and fibrosis induced by pressure overload, whereas either global- or cardiomyocyte-specific IRF8 knockout aggravated adverse remodeling (134). The investigators attributed this effect to an interaction between IRF8 and nuclear factor of activated T-cells, cytoplasmic 1 (NFATc1) which inhibits the nuclear translocation of NFATc1 (134). The authors speculated that the interaction between IRF8 and NFATc1 may prevent dephosphorylation of NFATc1 by calcineurin which ordinarily promotes nuclear translocation and facilitates pathological hypertrophy (134, 144).

#### IRF9

Lastly, Jiang et al. also studied the effects of IRF9 in the heart (135). In that study IRF9 protein levels were observed to be increased in the hearts of mice 2 and 4 weeks after aortic banding and in neonatal rat cardiomyocytes exposed to angiotensin II or isoproterenol (135). Cardiac-specific overexpression of IRF9 attenuated cardiac hypertrophy whereas hypertrophy, fibrosis and cardiac dysfunction were augmented in IRF9 knockout mice (135). These effects were attributed by the authors to an action of IRF9 in competing with p300 for binding to the transcription activation domain of myocardin (135), a transcriptional coactivator and inducer of cardiac hypertrophy (145).

### The IFN response and the heart in COVID-19

The COVID-19 pandemic has shone the spotlight on the role that IFNs play in the defence against viral infection, and potentially also the contribution of IFNs to adverse outcomes. COVID-19 can result in several different cardiovascular complications including heart failure, arrythmia, acute coronary syndrome, MI, myocarditis, and acute myocardial injury (146). Indeed, myocardial injury may be the most common extrapulmonary complication of COVID-19, affecting over 70% of those with severe disease (146). Most studies and commentaries discussing the role of IFNs in COVID-19 do not distinguish the cardiac effects specifically from systemic effects associated with severe disease or critical illness. Furthermore, whereas cardiac complications are common in severe COVID-19, the relative contributions of direct viral infection and the immune response to viral infection (including the IFN response) have not been disentangled. It is similarly challenging to disentangle the relative contributions of either individual IFNs or the IFN response from hyperinflammation in general (147, 148). Nevertheless, both a delayed persistent type I IFN response and diminished capacity to produce type I IFNs have been linked to COVID-19 severity (147). Thus, in COVID-19, the optimal IFN response is one that is finely balanced and dependent on host factors, stage and severity of disease and site of infection, amongst other factors (147, 148). For instance, a genome-wide association study has linked genes encoding members of IFN signaling pathways to critical illness in COVID-19 (149). However, Mendelian randomization revealed that life-threatening COVID-19 was associated with low expression of *IFNAR2*, but high expression of *TYK2* (149). Therapeutically, recombinant IFNβ1a did not reduce mortality in hospitalized patients with COVID-19 (150). In contrast, the anti-inflammatory therapy baricitinib, which has activity against JAK1/2 and moderate activity against TYK2, reduced mortality amongst hospitalized patients with COVID-19 by about 20%, and it has received U.S. Food and Drug Administration (FDA) approval for this indication (151). As discussed below, however, although JAK inhibitors may block IFN signaling, their effects are not limited to this pathway. In sum, the IFN response likely has a complex, bidirectional role in COVID-19, including the cardiac complications of COVID-19, both functioning in the host defence against viral infection, and contributing to the deleterious consequences of hyperinflammation in severe disease.

### IFNs and atherosclerosis

Both type I IFNs and IFN-γ have also been implicated in the pathogenesis of atherosclerosis. In brief, type I IFNs may affect plaque formation through several different processes, including through the formation of foam cells and macrophage extracellular traps, endothelial dysfunction and through influencing the actions of dendritic cells and T cells (152). IFN-γ affects cholesterol accumulation in macrophages and macrophage activation, induces foam cell formation and apoptosis, affects Th1-mediated immune responses, and promotes oxidative stress, endothelial activation, smooth muscle cell proliferation and plaque development (153, 154). Like its paradoxical actions in the heart, however, IFN-γ has been reported to have both pro- and anti-atherogenic effects (154). In this review, we have focused on the actions of IFNs in the heart and in heart disease. It should be noted that a comparably sized body of experimental evidence exists outlining the roles of both type I IFNs and IFN-γ in atherosclerosis. For an in-depth exposition, the reader is referred to reviews specifically on this topic (152–154).

### IFNs as prognostic markers

Whereas IFNs and interferon-related pathways play several different roles in heart disease, evidence supporting circulating IFNs as biomarkers of cardiac disease is scant. Those data that do exist support a greater role for the measurement of plasma levels of IFN-γ than for type I IFNs. For instance, in a study of patients with HCM, increased plasma levels of IFN-γ were associated with LV wall thickness (155) (Figure 5). Furthermore, lower circulating levels of IFN-γ have been associated with greater likelihood of reverse remodeling following LVAD implantation (116), and of less severe peripartum cardiomyopathy (156) (Figure 5). For type I IFNs, plasma proteomic analysis revealed that higher circulating levels of IFNA5 were associated with a higher relative wall thickness in women, but not in men (157). It may be that determination of a type I IFN signature, as has been done in SLE for instance (42, 158), may offer greater prognostic value than measurement of individual IFNs. However, even amongst patients with SLE who often exhibit a strong type I IFN response, a type I IFN signature has not been robustly associated with CVD (44). In sum, extensive experimental evidence and correlative clinical studies support roles of IFNs and IFN-related pathways in the pathogenesis of heart disease (or protection against it). However, there is an absence of evidence that would suggest routine measurement of IFNs (or their downstream effectors) will offer utility as biomarkers of cardiac disease, certainly above already established measures, and even amongst at-risk groups.

### Therapeutically targeting IFNs to improve outcomes in heart disease

To date, several different therapeutic approaches that augment or inhibit IFN pathways have received regulatory authority approval, although not for the treatment of heart disease. Most notably, these approaches include recombinant IFNs and small molecule JAK inhibitors. Elsewhere, there is fervent medicinal chemistry activity in the development of agents that interfere (or augment) cGAS-STING signaling, and newer strategies to modulate the actions of cytokines are under development.

#### Recombinant IFNs

As discussed earlier, recombinant IFNβ1b and recombinant IFNα2b are approved for other indications, including multiple sclerosis, chronic hepatitis, and hematological malignancy. Whereas clinical studies have suggested improvements in some outcomes for patients treated with recombinant IFN in viral myocarditis (159–162), recombinant IFN therapy is not part of usual viral myocarditis management, with somewhat disappointing trial results attributed possibly to a relatively poor response to IFN of parvovirus B1 and HHV6 myocarditis (163). Similarly, as already discussed, recombinant interferon IFNβ1a did not affect mortality in hospitalized patients with COVID-19 (150).

#### JAK inhibitors

Whereas the goal of recombinant IFN therapy is to augment signaling through IFN pathways, JAK inhibitors block IFN signaling. These therapies have received regulatory approval principally for the treatment of inflammatory arthropathies, and they have shown promise in other disease settings associated with inflammation or an augmented IFN response. However, by virtue of blocking JAK/STAT signaling, the effects of these agents extend to antagonizing the actions of several proinflammatory cytokines whose receptors signal through this pathway and they are not limited to antagonizing IFNs or IFN-related pathways. Tofacitinib is a JAK1/3 inhibitor, with less efficacy vs. JAK2 and TYK2, and it is approved for the treatment of rheumatoid arthritis, psoriatic arthritis and ulcerative colitis (164). Baricitinib is an inhibitor of JAK1/2, with moderate activity vs. TYK2, with an indication for the treatment of rheumatoid arthritis (165). As already discussed, baricitinib has also received FDA approval for use in the treatment of COVID-19. Upadacitinib is selective for JAK1 and is approved for the treatment of rheumatoid arthritis, psoriatic arthropathy, axial spondyloarthritis, atopic dermatitis, ulcerative dermatitis and Crohn's disease (166). In a small clinical trial, baricitinib was shown to improve disease severity amongst patients with monogenic IFN-mediated autoinflammatory diseases [including chronic atypical neutrophilic dermatosis with lipodystrophy and elevated temperatures (CANDLE), SAVI, and other interferonopathies] (167). Baricitinib also reduced albuminuria and improved inflammatory markers in patients with diabetic kidney disease (168). However, the product monograph for baricitinib carries warnings as to a potential increased risk of infection, malignancy, major adverse cardiovascular events, and thrombosis (165). Accordingly, whereas JAK inhibitors offer a repurposing opportunity, their adverse effect profiles may preclude their use in the treatment of heart disease.

#### Strategies that antagonize cGAS-STING signaling

The development of inhibitors of cGAS or STING (70), or of TBK1 (169), is an area of active investigation from both the academic and the industrial sectors. Over 20 compounds have already been reported to have cGAS- or STING-inhibitory effects (70), and activators of the cGAS-STING pathway are being developed as cancer therapeutics (170, 171). cGAS inhibitors are being developed that exert their effects by either blocking the catalytic site of cGAS (e.g., RU.521, Compound S3, G150, PF-06928125) or by interfering with cGAS binding to DNA (e.g., suramin, oligonucleotides, antimalarials) (70). Antagonists of STING have been developed that exert their effects by targeting the cGAMP binding site of STING (e.g., Astin C, tetrahydroisoquinolones) or by targeting STING palmitoylation which is necessary for its activation [e.g., indole ureas (especially H-151), nitrofurans, acrylamides, and nitro fatty acids] (70). Given the wealth of preclinical data attesting to the importance of cGAS-STING in the pathogenesis of cardiac disease, it will be interesting to see if any of these medicinal chemistry breakthroughs reach the clinical trial arena for this indication.

#### Future strategies to modulate signaling by IFNs and IFN-related pathways

Looking ahead, several different strategies are being trialled to either augment or antagonize cytokine action. The reader is directed to an excellent recent review on this topic (172). Briefly, various approaches are being developed to augment cytokine activity including the use of fusion proteins, PEGylation, polymeric matrices, microparticles, immune complexes and immunocytokines, orthogonal cytokines, mutagenesis, neokines, and surrogate agonists (172). Strategies to antagonize cytokine signaling that are under development include antibodies to cytokines or their receptors, JAK inhibitors, STAT inhibitors, proteolysis-targeting chimeras (PROTACs) and cytokine receptor targeting chimeras (or KineTACs) (172).

## Summary and future directions

In this review, we have considered the actions of type I IFNs and ISGs, cGAS-STING pathway activation, IFN-γ, and IRFs in the heart. From the body of experimental evidence, it is clear that each of these biological processes plays important roles in either protection against heart disease or in the pathogenesis of diseases of the heart. How might it be possible to exploit these discoveries for the benefit of patients?

It is unlikely that measurement of plasma IFNs, certainly single IFNs, will offer prognostic value in the management of heart disease. Alternatively though, within the biomedical research space, recent years have witnessed enormous advances in single cell technologies. These single cell technologies, and in particular scRNA-seq, are especially helpful in defining immune cell subpopulations (173), and identification of immune cell subpopulations that are enriched for IFN-related genes has been important to several recent studies elucidating the molecular pathological basis of cardiac diseases (37, 102, 174–180). Fundamental studies exploiting single cell technologies and defining immune cell subpopulations based on IFN-related gene enrichment are likely to continue to advance our understanding of the role of inflammation in heart disease in years ahead.

Therapeutically, various strategies may be employed to dampen signaling by IFNs or IFN-related pathway signaling. However, immunosuppressive therapies may be associated with an increased risk of malignancy and infection. For prevalent diseases such as heart disease, where therapies that improve outcomes already exist, the challenge will be the development of a therapeutic strategy that has an acceptable side effect profile. In this respect, the therapeutic targeting of a downstream pathway, such as cGAS-STING signaling, may offer theoretical advantages in that it leaves other immune defense pathways intact (70). Alternatively, a niche for therapies that antagonize IFN-related pathways may be found in the acute setting. Elsewhere, there has been renewed interest in the complex pathobiology of ISGs, and especially ISG15, since the emergence of SARS-CoV-2, and the recognition that ISG15 plays a key role in the defence against viral infection and in the immune response to viral infection (65). This renewed interest could pave the way for therapeutic advances for chronic diseases, such as heart disease, in the future. The biology of ISG15 is complex, the protein exerting different effects in its intracellular free or conjugated forms, and as a secreted protein, with species-specific differences between mice and humans (181). Similarly, it appears that IFN-γ can exert both beneficial and detrimental effects on the heart. However, just because the biology may be complicated, does not mean that it is not important.

In summary, IFNs and IFN-related pathways play important roles in the inflammation that commonly accompanies diseases of the heart. These roles are complex, being dependent on the nature of the underlying cardiac insult, the pathway itself, stage of disease, and host factors. As technological breakthroughs continue to advance the study of fundamental biology, a more nuanced understanding of the actions of IFNs and IFN-related pathway is sure to follow. Whether these advances will ultimately lead to improved outcomes for those affected by heart disease awaits to be seen.

## References

[1] Isaacs A Lindenmann J. Virus interference. I. the Interferon. Proc R Soc Lond B Biol Sci. (1957) 147:258–67. 10.1098/rspb.1957.0048 26297790

[2] Renauld JC. Class II cytokine receptors and their ligands: key antiviral and inflammatory modulators. Nat Rev Immunol. (2003) 3:667–76. 10.1038/nri1153 12974481

[3] Pestka S Krause CD Walter MR. Interferons, interferon-like cytokines, and their receptors. Immunol Rev. (2004) 202:8–32. 10.1111/j.0105-2896.2004.00204.x 15546383

[4] Mcnab F Mayer-Barber K Sher A Wack A O'garra A. Type I interferons in infectious disease. Nat Rev Immunol. (2015) 15:87–103. 10.1038/nri3787 25614319 PMC7162685

[5] Negishi H Taniguchi T Yanai H. The interferon (IFN) class of cytokines and the IFN regulatory factor (IRF) transcription factor family. Cold Spring Harb Perspect Biol. (2018) 10:a028423. 10.1101/cshperspect.a028423 28963109 PMC6211389

[6] Hervas-Stubbs S Perez-Gracia JL Rouzaut A Sanmamed MF Le Bon A Melero I. Direct effects of type I interferons on cells of the immune system. Clin Cancer Res. (2011) 17:2619–27. 10.1158/1078-0432.CCR-10-1114 21372217

[7] Chow KT Gale M Jr. Snapshot: interferon signaling. Cell. (2015) 163:1808–e1801. 10.1016/j.cell.2015.12.008 26687364

[8] Platanias LC. Mechanisms of type-I- and type-II-interferon-mediated signalling. Nat Rev Immunol. (2005) 5:375–86. 10.1038/nri1604 15864272

[9] Sheppard P Kindsvogel W Xu W Henderson K Schlutsmeyer S Whitmore TE IL-28, IL-29 and their class II cytokine receptor IL-28R. Nat Immunol. (2003) 4:63–8. 10.1038/ni873 12469119

[10] Iversen MB Paludan SR. Mechanisms of type III interferon expression. J Interferon Cytokine Res. (2010) 30:573–8. 10.1089/jir.2010.0063 20645874

[11] Kawasaki T Kawai T. Toll-like receptor signaling pathways. Front Immunol. (2014) 5:461. 10.3389/fimmu.2014.00461 25309543 PMC4174766

[12] Hagen SH Henseling F Hennesen J Savel H Delahaye S Richert L Heterogeneous escape from X chromosome inactivation results in sex differences in type I IFN responses at the single human pDC level. Cell Rep. (2020) 33:108485. 10.1016/j.celrep.2020.108485 33296655 PMC7833293

[13] Yoneyama M Kikuchi M Matsumoto K Imaizumi T Miyagishi M Taira K Shared and unique functions of the DExD/H-box helicases RIG-I, MDA5, and LGP2 in antiviral innate immunity. J Immunol. (2005) 175:2851–8. 10.4049/jimmunol.175.5.2851 16116171

[14] Kato H Takeuchi O Sato S Yoneyama M Yamamoto M Matsui K Differential roles of MDA5 and RIG-I helicases in the recognition of RNA viruses. Nature. (2006) 441:101–5. 10.1038/nature04734 16625202

[15] Seth RB Sun L Ea CK Chen ZJ. Identification and characterization of MAVS, a mitochondrial antiviral signaling protein that activates NF-kappaB and IRF 3. Cell. (2005) 122:669–82. 10.1016/j.cell.2005.08.012 16125763

[16] Zhong B Yang Y Li S Wang YY Li Y Diao F The adaptor protein MITA links virus-sensing receptors to IRF3 transcription factor activation. Immunity. (2008) 29:538–50. 10.1016/j.immuni.2008.09.003 18818105

[17] Ishikawa H Ma Z Barber GN. STING Regulates intracellular DNA-mediated, type I interferon-dependent innate immunity. Nature. (2009) 461:788–92. 10.1038/nature08476 19776740 PMC4664154

[18] Shemesh M Lochte S Piehler J Schreiber G. IFNAR1 and IFNAR2 play distinct roles in initiating type I interferon-induced JAK-STAT signaling and activating STATs. Sci Signal. (2021) 14:eabe4627. 10.1126/scisignal.abe4627 34813358

[19] Decker T Muller M Stockinger S. The yin and yang of type I interferon activity in bacterial infection. Nat Rev Immunol. (2005) 5:675–87. 10.1038/nri1684 16110316

[20] Uze G Monneron D. IL-28 and IL-29: newcomers to the interferon family. Biochimie. (2007) 89:729–34. 10.1016/j.biochi.2007.01.008 17367910

[21] Lazear HM Nice TJ Diamond MS. Interferon-lambda: immune functions at barrier surfaces and beyond. Immunity. (2015) 43:15–28. 10.1016/j.immuni.2015.07.001 26200010 PMC4527169

[22] Liu S Cai X Wu J Cong Q Chen X Li T Phosphorylation of innate immune adaptor proteins MAVS, STING, and TRIF induces IRF3 activation. Science. (2015) 347:aaa2630. 10.1126/science.aaa2630 25636800

[23] Hida S Ogasawara K Sato K Abe M Takayanagi H Yokochi T CD8(+) T cell-mediated skin disease in mice lacking IRF-2, the transcriptional attenuator of interferon-alpha/beta signaling. Immunity. (2000) 13:643–55. 10.1016/S1074-7613(00)00064-9 11114377

[24] Schoggins JW Rice CM. Interferon-stimulated genes and their antiviral effector functions. Curr Opin Virol. (2011) 1:519–25. 10.1016/j.coviro.2011.10.008 22328912 PMC3274382

[25] Williams BR. PKR; a sentinel kinase for cellular stress. Oncogene. (1999) 18:6112–20. 10.1038/sj.onc.1203127 10557102

[26] Rahnefeld A Klingel K Schuermann A Diny NL Althof N Lindner A Ubiquitin-like protein ISG15 (interferon-stimulated gene of 15 kDa) in host defense against heart failure in a mouse model of virus-induced cardiomyopathy. Circulation. (2014) 130:1589–600. 10.1161/CIRCULATIONAHA.114.009847 25165091

[27] Yerra VG Batchu SN Kaur H Kabir MDG Liu Y Advani SL Pressure overload induces ISG15 to facilitate adverse ventricular remodeling and promote heart failure. J Clin Invest. (2023) 133:e161453. 10.1172/JCI161453 37115698 PMC10145941

[28] Dolei A Capobianchi MR Ameglio F. Human interferon-gamma enhances the expression of class I and class II major histocompatibility complex products in neoplastic cells more effectively than interferon-alpha and interferon-beta. Infect Immun. (1983) 40:172–6. 10.1128/iai.40.1.172-176.1983 6299957 PMC264832

[29] Greiner JW Hand PH Noguchi P Fisher PB Pestka S Schlom J. Enhanced expression of surface tumor-associated antigens on human breast and colon tumor cells after recombinant human leukocyte alpha-interferon treatment. Cancer Res. (1984) 44:3208–14. 6744259

[30] Clemens MJ. Interferons and apoptosis. J Interferon Cytokine Res. (2003) 23:277–92. 10.1089/107999003766628124 12859854

[31] Takaoka A Hayakawa S Yanai H Stoiber D Negishi H Kikuchi H Integration of interferon-alpha/beta signalling to p53 responses in tumour suppression and antiviral defence. Nature. (2003) 424:516–23. 10.1038/nature01850 12872134

[32] Moiseeva O Mallette FA Mukhopadhyay UK Moores A Ferbeyre G. DNA damage signaling and p53-dependent senescence after prolonged beta-interferon stimulation. Mol Biol Cell. (2006) 17:1583–92. 10.1091/mbc.e05-09-0858 16436515 PMC1415317

[33] Keay S Grossberg SE. Interferon inhibits the conversion of 3T3-L1 mouse fibroblasts into adipocytes. Proc Natl Acad Sci U S A. (1980) 77:4099–103. 10.1073/pnas.77.7.4099 6159626 PMC349777

[34] Fidler IJ. Regulation of neoplastic angiogenesis. J Natl Cancer Inst Monogr. (2001) 28:10–4. 10.1093/oxfordjournals.jncimonographs.a024251 11158201

[35] Dunn GP Bruce AT Sheehan KC Shankaran V Uppaluri R Bui JD A critical function for type I interferons in cancer immunoediting. Nat Immunol. (2005) 6:722–9. 10.1038/ni1213 15951814

[36] Borden EC Sen GC Uze G Silverman RH Ransohoff RM Foster GR Interferons at age 50: past, current and future impact on biomedicine. Nat Rev Drug Discov. (2007) 6:975–90. 10.1038/nrd2422 18049472 PMC7097588

[37] King KR Aguirre AD Ye YX Sun Y Roh JD Ng RP Jr IRF3 and type I interferons fuel a fatal response to myocardial infarction. Nat Med. (2017) 23:1481–7. 10.1038/nm.4428 29106401 PMC6477926

[38] Althof N Harkins S Kemball CC Flynn CT Alirezaei M Whitton JL. *In vivo* ablation of type I interferon receptor from cardiomyocytes delays coxsackieviral clearance and accelerates myocardial disease. J Virol. (2014) 88:5087–99. 10.1128/JVI.00184-14 24574394 PMC3993796

[39] Liu A Ying S. Aicardi-goutieres syndrome: a monogenic type I interferonopathy. Scand J Immunol. (2023) 98:e13314. 10.1111/sji.13314 37515439

[40] Crow YJ Chase DS Lowenstein Schmidt J Szynkiewicz M Forte GM Gornall HL Characterization of human disease phenotypes associated with mutations in TREX1, RNASEH2A, RNASEH2B, RNASEH2C, SAMHD1, ADAR, and IFIH1. Am J Med Genet A. (2015) 167A:296–312. 10.1002/ajmg.a.36887 25604658 PMC4382202

[41] Ambrosi A Thorlacius GE Sonesson SE Wahren-Herlenius M. Interferons and innate immune activation in autoimmune congenital heart block. Scand J Immunol. (2021) 93:e12995. 10.1111/sji.12995 33188653

[42] Vital EM Merrill JT Morand EF Furie RA Bruce IN Tanaka Y Anifrolumab efficacy and safety by type I interferon gene signature and clinical subgroups in patients with SLE: post hoc analysis of pooled data from two phase III trials. Ann Rheum Dis. (2022) 81:951–61. 10.1136/annrheumdis-2021-221425 35338035 PMC9213795

[43] Frostegard J. Systemic lupus erythematosus and cardiovascular disease. J Intern Med. (2023) 293:48–62. 10.1111/joim.13557 35982610 PMC10087345

[44] Kirchler C Husar-Memmer E Rappersberger K Thaler K Fritsch-Stork R. Type I interferon as cardiovascular risk factor in systemic and cutaneous lupus erythematosus: a systematic review. Autoimmun Rev. (2021) 20:102794. 10.1016/j.autrev.2021.102794 33722754

[45] Anonymous. Available online at: https://health-products.canada.ca/dpd-bdpp/info?lang=eng&code=19241 (accessed October 19, 2023) (2023a).

[46] Anonymous. Available online at: https://www.merck.ca/en/wp-content/uploads/sites/20/2021/04/INTRON_A-PM_E.pdf (accessed October 19, 2023) (2023b).

[47] Wessely R Klingel K Knowlton KU Kandolf R. Cardioselective infection with coxsackievirus B3 requires intact type I interferon signaling: implications for mortality and early viral replication. Circulation. (2001) 103:756–61. 10.1161/01.CIR.103.5.756 11156890

[48] Deonarain R Cerullo D Fuse K Liu PP Fish EN. Protective role for interferon-beta in coxsackievirus B3 infection. Circulation. (2004) 110:3540–3. 10.1161/01.CIR.0000136824.73458.20 15249500

[49] Perng YC Lenschow DJ. ISG15 in antiviral immunity and beyond. Nat Rev Microbiol. (2018) 16:423–39. 10.1038/s41579-018-0020-5 29769653 PMC7097117

[50] Bogunovic D Byun M Durfee LA Abhyankar A Sanal O Mansouri D Mycobacterial disease and impaired IFN-γ immunity in humans with inherited ISG15 deficiency. Science. (2012) 337:1684–8. 10.1126/science.1224026 22859821 PMC3507439

[51] Swaim CD Scott AF Canadeo LA Huibregtse JM. Extracellular ISG15 signals cytokine secretion through the LFA-1 integrin receptor. Mol Cell. (2017) 68:581–90.e585. 10.1016/j.molcel.2017.10.003 29100055 PMC5690536

[52] Bredow C Thery F Wirth EK Ochs S Kespohl M Kleinau G ISG15 blocks cardiac glycolysis and ensures sufficient mitochondrial energy production during coxsackievirus B3 infection. Cardiovasc Res. (2024) Epub ahead of print Feb 3. 10.1093/cvr/cvae026 38309955 PMC11074791

[53] Maier HJ Schips TG Wietelmann A Kruger M Brunner C Sauter M Cardiomyocyte-specific IkappaB kinase (IKK)/NF-kappaB activation induces reversible inflammatory cardiomyopathy and heart failure. Proc Natl Acad Sci U S A. (2012) 109:11794–9. 10.1073/pnas.1116584109 22753500 PMC3406816

[54] Zhao C Denison C Huibregtse JM Gygi S Krug RM. Human ISG15 conjugation targets both IFN-induced and constitutively expressed proteins functioning in diverse cellular pathways. Proc Natl Acad Sci U S A. (2005) 102:10200–5. 10.1073/pnas.0504754102 16009940 PMC1177427

[55] Gough DJ Messina NL Clarke CJ Johnstone RW Levy DE. Constitutive type I interferon modulates homeostatic balance through tonic signaling. Immunity. (2012) 36:166–74. 10.1016/j.immuni.2012.01.011 22365663 PMC3294371

[56] Malakhov MP Kim KI Malakhova OA Jacobs BS Borden EC Zhang DE. High-throughput immunoblotting. Ubiquitiin-like protein ISG15 modifies key regulators of signal transduction. J Biol Chem. (2003) 278:16608–13. 10.1074/jbc.M208435200 12582176

[57] Durfee LA Huibregtse JM. The ISG15 conjugation system. Methods Mol Biol. (2012) 832:141–9. 10.1007/978-1-61779-474-2_9 22350882 PMC5912894

[58] Albert M Becares M Falqui M Fernandez-Lozano C Guerra S. ISG15, A small molecule with huge implications: regulation of mitochondrial homeostasis. Viruses. (2018) 10:629. 10.3390/v10110629 30428561 PMC6265978

[59] Ying X Zhao Y Yao T Yuan A Xu L Gao L Novel protective role for ubiquitin-specific protease 18 in pathological cardiac remodeling. Hypertension. (2016) 68:1160–70. 10.1161/HYPERTENSIONAHA.116.07562 27572150

[60] Liu X Li H Zhong B Blonska M Gorjestani S Yan M USP18 inhibits NF-kappaB and NFAT activation during Th17 differentiation by deubiquitinating the TAK1-TAB1 complex. J Exp Med. (2013) 210:1575–90. 10.1084/jem.20122327 23825189 PMC3727316

[61] Wang C Deng L Hong M Akkaraju GR Inoue J Chen ZJ. TAK1 is a ubiquitin-dependent kinase of MKK and IKK. Nature. (2001) 412:346–51. 10.1038/35085597 11460167

[62] Basters A Geurink PP El Oualid F Ketscher L Casutt MS Krause E Molecular characterization of ubiquitin-specific protease 18 reveals substrate specificity for interferon-stimulated gene 15. FEBS J. (2014) 281:1918–28. 10.1111/febs.12754 24533902

[63] Basters A Geurink PP Rocker A Witting KF Tadayon R Hess S Structural basis of the specificity of USP18 toward ISG15. Nat Struct Mol Biol. (2017) 24:270–8. 10.1038/nsmb.3371 28165509 PMC5405867

[64] Farrell PJ Broeze RJ Lengyel P. Accumulation of an mRNA and protein in interferon-treated ehrlich ascites tumour cells. Nature. (1979) 279:523–5. 10.1038/279523a0 571963

[65] Sarkar L Liu G Gack MU. ISG15: its roles in SARS-CoV-2 and other viral infections. Trends Microbiol. (2023) 31:1262–75. 10.1016/j.tim.2023.07.006 37573184 PMC10840963

[66] Sun L Wu J Du F Chen X Chen ZJ. Cyclic GMP-AMP synthase is a cytosolic DNA sensor that activates the type I interferon pathway. Science. (2013) 339:786–91. 10.1126/science.1232458 23258413 PMC3863629

[67] Wu J Sun L Chen X Du F Shi H Chen C Cyclic GMP-AMP is an endogenous second messenger in innate immune signaling by cytosolic DNA. Science. (2013) 339:826–30. 10.1126/science.1229963 23258412 PMC3855410

[68] Oduro PK Zheng X Wei J Yang Y Wang Y Zhang H The cGAS-STING signaling in cardiovascular and metabolic diseases: future novel target option for pharmacotherapy. Acta Pharm Sin B. (2022) 12:50–75. 10.1016/j.apsb.2021.05.011 35127372 PMC8799861

[69] Tanaka Y Chen ZJ. STING specifies IRF3 phosphorylation by TBK1 in the cytosolic DNA signaling pathway. Sci Signal. (2012) 5:ra20. 10.1126/scisignal.2002521 22394562 PMC3549669

[70] Decout A Katz JD Venkatraman S Ablasser A. The cGAS-STING pathway as a therapeutic target in inflammatory diseases. Nat Rev Immunol. (2021) 21:548–69. 10.1038/s41577-021-00524-z 33833439 PMC8029610

[71] Balka KR Louis C Saunders TL Smith AM Calleja DJ D’silva DB TBK1 and IKKepsilon act redundantly to mediate STING-induced NF-kappaB responses in myeloid cells. Cell Rep. (2020) 31:107492. 10.1016/j.celrep.2020.03.056 32268090

[72] Yum S Li M Fang Y Chen ZJ. TBK1 recruitment to STING activates both IRF3 and NF-kappaB that mediate immune defense against tumors and viral infections. Proc Natl Acad Sci U S A. (2021) 118:e2100225118. 10.1073/pnas.2100225118 33785602 PMC8040795

[73] Gui X Yang H Li T Tan X Shi P Li M Autophagy induction via STING trafficking is a primordial function of the cGAS pathway. Nature. (2019) 567:262–6. 10.1038/s41586-019-1006-9 30842662 PMC9417302

[74] Gaidt MM Ebert TS Chauhan D Ramshorn K Pinci F Zuber S The DNA inflammasome in human myeloid cells is initiated by a STING-cell death program upstream of NLRP3. Cell. (2017) 171:1110–24 e1118. 10.1016/j.cell.2017.09.039 29033128 PMC5901709

[75] Wan W Qian C Wang Q Li J Zhang H Wang L STING directly recruits WIPI2 for autophagosome formation during STING-induced autophagy. EMBO J. (2023) 42:e112387. 10.15252/embj.2022112387 36872914 PMC10106988

[76] Liu B Carlson RJ Pires IS Gentili M Feng E Hellier Q Human STING is a proton channel. Science. (2023) 381:508–14. 10.1126/science.adf8974 37535724 PMC11260435

[77] Cao DJ Schiattarella GG Villalobos E Jiang N May HI Li T Cytosolic DNA sensing promotes macrophage transformation and governs myocardial ischemic injury. Circulation. (2018) 137:2613–34. 10.1161/CIRCULATIONAHA.117.031046 29437120 PMC5997506

[78] Hu D Cui YX Wu MY Li L Su LN Lian Z Cytosolic DNA sensor cGAS plays an essential pathogenetic role in pressure overload-induced heart failure. Am J Physiol Heart Circ Physiol. (2020) 318:H1525–37. 10.1152/ajpheart.00097.2020 32383996

[79] Gong Y Li G Tao J Wu NN Kandadi MR Bi Y Double knockout of Akt2 and AMPK accentuates high fat diet-induced cardiac anomalies through a cGAS-STING-mediated mechanism. Biochim Biophys Acta Mol Basis Dis. (2020) 1866:165855. 10.1016/j.bbadis.2020.165855 32512189

[80] Wang S Wang L Qin X Turdi S Sun D Culver B ALDH2 contributes to melatonin-induced protection against APP/PS1 mutation-prompted cardiac anomalies through cGAS-STING-TBK1-mediated regulation of mitophagy. Signal Transduct Target Ther. (2020) 5:119. 10.1038/s41392-020-0171-5 32703954 PMC7378833

[81] Liu F Liu Y Zhuang Z Ma J Xu X Zhang W Beclin1 haploinsufficiency accentuates second-hand smoke exposure—induced myocardial remodeling and contractile dysfunction through a STING-mediated mechanism. J Mol Cell Cardiol. (2020) 148:78–88. 10.1016/j.yjmcc.2020.08.016 32891637

[82] Rech L Abdellatif M Pottler M Stangl V Mabotuwana N Hardy S Small molecule STING inhibition improves myocardial infarction remodeling. Life Sci. (2022) 291:120263. 10.1016/j.lfs.2021.120263 34971697

[83] Hu S Gao Y Gao R Wang Y Qu Y Yang J The selective STING inhibitor H-151 preserves myocardial function and ameliorates cardiac fibrosis in murine myocardial infarction. Int Immunopharmacol. (2022) 107:108658. 10.1016/j.intimp.2022.108658 35278833

[84] Yan M Li Y Luo Q Zeng W Shao X Li L Mitochondrial damage and activation of the cytosolic DNA sensor cGAS-STING pathway lead to cardiac pyroptosis and hypertrophy in diabetic cardiomyopathy mice. Cell Death Discov. (2022) 8:258. 10.1038/s41420-022-01046-w 35538059 PMC9091247

[85] Xiao Z Yu Z Chen C Chen R Su Y. GAS-STING signaling plays an essential pathogenetic role in doxorubicin-induced cardiotoxicity. BMC Pharmacol Toxicol. (2023) 24:19. 10.1186/s40360-022-00631-0 36964634 PMC10037834

[86] Wu Z Miao X Jiang Y Kong D Liu H Xie W Cardiomyocytic cyclic GMP-AMP synthase is critical for the induction of experimental cardiac graft rejection. J Thorac Cardiovasc Surg. (2023b) 166:e406–27. 10.1016/j.jtcvs.2023.03.005 37061907

[87] Luo W Zou X Wang Y Dong Z Weng X Pei Z Critical role of the cGAS-STING pathway in doxorubicin-induced cardiotoxicity. Circ Res. (2023) 132:e223–42. 10.1161/CIRCRESAHA.122.321369 37154056

[88] Lei Y Vanportfliet JJ Chen YF Bryant JD Li Y Fails D Cooperative sensing of mitochondrial DNA by ZBP1 and cGAS promotes cardiotoxicity. Cell. (2023) 186:3013–32 e3022. 10.1016/j.cell.2023.05.039 37352855 PMC10330843

[89] Mao H Angelini A Li S Wang G Li L Patterson C CRAT links cholesterol metabolism to innate immune responses in the heart. Nat Metab. (2023) 5:1382–94. 10.1038/s42255-023-00844-5 37443356 PMC10685850

[90] Zhang Y Chen W Wang Y. STING is an essential regulator of heart inflammation and fibrosis in mice with pathological cardiac hypertrophy via endoplasmic reticulum (ER) stress. Biomed Pharmacother. (2020) 125:110022. 10.1016/j.biopha.2020.110022 32106379

[91] Li N Zhou H Wu H Wu Q Duan M Deng W STING-IRF3 contributes to lipopolysaccharide-induced cardiac dysfunction, inflammation, apoptosis and pyroptosis by activating NLRP3. Redox Biol. (2019) 24:101215. 10.1016/j.redox.2019.101215 31121492 PMC6529775

[92] Han YL Li YL Jia LX Cheng JZ Qi YF Zhang HJ Reciprocal interaction between macrophages and T cells stimulates IFN-gamma and MCP-1 production in Ang II-induced cardiac inflammation and fibrosis. PLoS One. (2012) 7:e35506. 10.1371/journal.pone.0035506 22567105 PMC3342394

[93] Ong S Rose NR Cihakova D. Natural killer cells in inflammatory heart disease. Clin Immunol. (2017) 175:26–33. 10.1016/j.clim.2016.11.010 27894980 PMC5315604

[94] Kimura A Ishida Y Furuta M Nosaka M Kuninaka Y Taruya A Protective roles of interferon-gamma in cardiac hypertrophy induced by sustained pressure overload. J Am Heart Assoc. (2018) 7:e008145. 10.1161/JAHA.117.008145 29555642 PMC5907566

[95] Levick SP Goldspink PH. Could interferon-gamma be a therapeutic target for treating heart failure? Heart Fail Rev. (2014) 19:227–36. 10.1007/s10741-013-9393-8 23589353 PMC3844057

[96] Mccarthy MK Procario MC Twisselmann N Wilkinson JE Archambeau AJ Michele DE Proinflammatory effects of interferon gamma in mouse adenovirus 1 myocarditis. J Virol. (2015) 89:468–79. 10.1128/JVI.02077-14 25320326 PMC4301126

[97] Cunha-Neto E Dzau VJ Allen PD Stamatiou D Benvenutti L Higuchi ML Cardiac gene expression profiling provides evidence for cytokinopathy as a molecular mechanism in Chagas’ disease cardiomyopathy. Am J Pathol. (2005) 167:305–13. 10.1016/S0002-9440(10)62976-8 16049318 PMC1603558

[98] Rocha Rodrigues DB Dos Reis MA Romano A Pereira SA Teixeira Vde P Tostes S Jr *In situ* expression of regulatory cytokines by heart inflammatory cells in Chagas’ disease patients with heart failure. Clin Dev Immunol. (2012) 2012:361730. 10.1155/2012/361730 22811738 PMC3397162

[99] Nunes JPS Andrieux P Brochet P Almeida RR Kitano E Honda AK Co-exposure of cardiomyocytes to IFN-gamma and TNF-alpha induces mitochondrial dysfunction and nitro-oxidative stress: implications for the pathogenesis of chronic chagas disease cardiomyopathy. Front Immunol. (2021) 12:755862. 10.3389/fimmu.2021.755862 34867992 PMC8632642

[100] Finger S Knorr M Molitor M Schuler R Garlapati V Waisman A A sequential interferon gamma directed chemotactic cellular immune response determines survival and cardiac function post-myocardial infarction. Cardiovasc Res. (2019) 115:1907–17. 10.1093/cvr/cvz092 30949687

[101] Levick SP Mclarty JL Murray DB Freeman RM Carver WE Brower GL. Cardiac mast cells mediate left ventricular fibrosis in the hypertensive rat heart. Hypertension. (2009) 53:1041–7. 10.1161/HYPERTENSIONAHA.108.123158 19398662

[102] Ashour D Rebs S Arampatzi P Saliba AE Dudek J Schulz R An interferon gamma response signature links myocardial aging and immunosenescence. Cardiovasc Res. (2023) 119:2458–68. 10.1093/cvr/cvad068 37141306 PMC10651211

[103] Sun X Delbridge LM Dusting GJ. Cardiodepressant effects of interferon-gamma and endotoxin reversed by inhibition of NO synthase 2 in rat myocardium. J Mol Cell Cardiol. (1998) 30:989–97. 10.1006/jmcc.1998.0663 9618239

[104] Rosas GO Zieman SJ Donabedian M Vandegaer K Hare JM. Augmented age-associated innate immune responses contribute to negative inotropic and lusitropic effects of lipopolysaccharide and interferon gamma. J Mol Cell Cardiol. (2001) 33:1849–59. 10.1006/jmcc.2001.1448 11603926

[105] Reifenberg K Lehr HA Torzewski M Steige G Wiese E Kupper I Interferon-gamma induces chronic active myocarditis and cardiomyopathy in transgenic mice. Am J Pathol. (2007) 171:463–72. 10.2353/ajpath.2007.060906 17556594 PMC1934522

[106] Torzewski M Wenzel P Kleinert H Becker C El-Masri J Wiese E Chronic inflammatory cardiomyopathy of interferon gamma-overexpressing transgenic mice is mediated by tumor necrosis factor-alpha. Am J Pathol. (2012) 180:73–81. 10.1016/j.ajpath.2011.09.006 22051774

[107] Cosper PF Harvey PA Leinwand LA. Interferon-gamma causes cardiac myocyte atrophy via selective degradation of myosin heavy chain in a model of chronic myocarditis. Am J Pathol. (2012) 181:2038–46. 10.1016/j.ajpath.2012.08.040 23058369 PMC3509765

[108] Eriksson U Kurrer MO Bingisser R Eugster HP Saremaslani P Follath F Lethal autoimmune myocarditis in interferon-gamma receptor-deficient mice: enhanced disease severity by impaired inducible nitric oxide synthase induction. Circulation. (2001) 103:18–21. 10.1161/01.CIR.103.1.18 11136679

[109] Norose K Mun HS Aosai F Chen M Hata H Tagawa Y Organ infectivity of toxoplasma gondii in interferon-gamma knockout mice. J Parasitol. (2001) 87:447–52. 10.1645/0022-3395(2001)087[0447:OIOTGI]2.0.CO;2 11318585

[110] Michailowsky V Silva NM Rocha CD Vieira LQ Lannes-Vieira J Gazzinelli RT. Pivotal role of interleukin-12 and interferon-gamma axis in controlling tissue parasitism and inflammation in the heart and central nervous system during Trypanosoma cruzi infection. Am J Pathol. (2001) 159:1723–33. 10.1016/S0002-9440(10)63019-2 11696433 PMC3277321

[111] Afanasyeva M Georgakopoulos D Fairweather D Caturegli P Kass DA Rose NR. Novel model of constrictive pericarditis associated with autoimmune heart disease in interferon-gamma-knockout mice. Circulation. (2004) 110:2910–7. 10.1161/01.CIR.0000147538.92263.3A 15505106

[112] Fairweather D Frisancho-Kiss S Yusung SA Barrett MA Davis SE Gatewood SJ Interferon-gamma protects against chronic viral myocarditis by reducing mast cell degranulation, fibrosis, and the profibrotic cytokines transforming growth factor-beta 1, interleukin-1 beta, and interleukin-4 in the heart. Am J Pathol. (2004) 165:1883–94. 10.1016/S0002-9440(10)63241-5 15579433 PMC1618717

[113] Jin H Li W Yang R Ogasawara A Lu H Paoni NF. Inhibitory effects of interferon-gamma on myocardial hypertrophy. Cytokine. (2005) 31:405–14. 10.1016/j.cyto.2005.06.013 16105741

[114] Garcia AG Wilson RM Heo J Murthy NR Baid S Ouchi N Interferon-gamma ablation exacerbates myocardial hypertrophy in diastolic heart failure. Am J Physiol Heart Circ Physiol. (2012) 303:H587–96. 10.1152/ajpheart.00298.2012 22730392 PMC3468473

[115] Yan X Qiu W Jia B Zhong H Li X Chen Z. Myocardial protection by interferon-gamma late preconditioning during cardiopulmonary bypass-associated myocardial ischemia-reperfusion in pigs. Oncol Rep. (2013) 30:2145–52. 10.3892/or.2013.2707 24002640

[116] Diakos NA Taleb I Kyriakopoulos CP Shah KS Javan H Richins TJ Circulating and myocardial cytokines predict cardiac structural and functional improvement in patients with heart failure undergoing mechanical circulatory support. J Am Heart Assoc. (2021) 10:e020238. 10.1161/JAHA.120.020238 34595931 PMC8751895

[117] Borda E Leiros CP Sterin-Borda L De Bracco MM. Cholinergic response of isolated rat atria to recombinant rat interferon-gamma. J Neuroimmunol. (1991) 32:53–9. 10.1016/0165-5728(91)90071-E 1900518

[118] Hellkvist J Tufveson G Gerdin B Johnsson C. Characterization of fibroblasts from rejecting tissue: the hyaluronan production is increased. Transplantation. (2002) 74:1672–7. 10.1097/00007890-200212270-00004 12499878

[119] Miyamoto M Fujita T Kimura Y Maruyama M Harada H Sudo Y Regulated expression of a gene encoding a nuclear factor, IRF-1, that specifically binds to IFN-beta gene regulatory elements. Cell. (1988) 54:903–13. 10.1016/S0092-8674(88)91307-4 3409321

[120] Xie RL Gupta S Miele A Shiffman D Stein JL Stein GS The tumor suppressor interferon regulatory factor 1 interferes with SP1 activation to repress the human CDK2 promoter. J Biol Chem. (2003) 278:26589–96. 10.1074/jbc.M301491200 12732645

[121] Harada H Fujita T Miyamoto M Kimura Y Maruyama M Furia A Structurally similar but functionally distinct factors, IRF-1 and IRF-2, bind to the same regulatory elements of IFN and IFN-inducible genes. Cell. (1989) 58:729–39. 10.1016/0092-8674(89)90107-4 2475256

[122] Jefferies CA. Regulating IRFs in IFN driven disease. Front Immunol. (2019) 10:325. 10.3389/fimmu.2019.00325 30984161 PMC6449421

[123] Hatada S Kinoshita M Takahashi S Nishihara R Sakumoto H Fukui A An interferon regulatory factor-related gene (xIRF-6) is expressed in the posterior mesoderm during the early development of Xenopus laevis. Gene. (1997) 203:183–8. 10.1016/S0378-1119(97)00512-X 9426249

[124] Jiang DS Li L Huang L Gong J Xia H Liu X Interferon regulatory factor 1 is required for cardiac remodeling in response to pressure overload. Hypertension. (2014a) 64:77–86. 10.1161/HYPERTENSIONAHA.114.03229 24732887

[125] Huang Y Wang S Zhou J Liu Y Du C Yang K IRF1-mediated downregulation of PGC1alpha contributes to cardiorenal syndrome type 4. Nat Commun. (2020) 11:4664. 10.1038/s41467-020-18519-0 32938919 PMC7494935

[126] Li Y Wang Y Guo H Wu Q Hu Y. IRF2 contributes to myocardial infarction via regulation of GSDMD induced pyroptosis. Mol Med Rep. (2022b) 25:40. 10.3892/mmr.2021.12556 34878155 PMC8674697

[127] Tsushima K Osawa T Yanai H Nakajima A Takaoka A Manabe I IRF3 regulates cardiac fibrosis but not hypertrophy in mice during angiotensin II-induced hypertension. FASEB J. (2011) 25:1531–43. 10.1096/fj.10-174615 21266535

[128] Lu J Bian ZY Zhang R Zhang Y Liu C Yan L Interferon regulatory factor 3 is a negative regulator of pathological cardiac hypertrophy. Basic Res Cardiol. (2013) 108:326. 10.1007/s00395-012-0326-9 23307144

[129] Jiang DS Bian ZY Zhang Y Zhang SM Liu Y Zhang R Role of interferon regulatory factor 4 in the regulation of pathological cardiac hypertrophy. Hypertension. (2013) 61:1193–202. 10.1161/HYPERTENSIONAHA.111.00614 23589561 PMC3734933

[130] Courties G Heidt T Sebas M Iwamoto Y Jeon D Truelove J *In vivo* silencing of the transcription factor IRF5 reprograms the macrophage phenotype and improves infarct healing. J Am Coll Cardiol. (2014) 63:1556–66. 10.1016/j.jacc.2013.11.023 24361318 PMC3992176

[131] Nie S Dong B Gao S Zhou Y Lu W Fang M The protective effect of interfering TLR9-IRF5 signaling pathway on the development of CVB3-induced myocarditis. Clin Immunol. (2019) 207:24–35. 10.1016/j.clim.2019.07.002 31279856

[132] Jiang DS Liu Y Zhou H Zhang Y Zhang XD Zhang XF Interferon regulatory factor 7 functions as a novel negative regulator of pathological cardiac hypertrophy. Hypertension. (2014b) 63:713–22. 10.1161/HYPERTENSIONAHA.113.02653 24396025 PMC5349187

[133] Garcia-Gonzalez C Dieterich C Maroli G Wiesnet M Wietelmann A Li X ADAR1 prevents autoinflammatory processes in the heart mediated by IRF7. Circ Res. (2022) 131:580–97. 10.1161/CIRCRESAHA.122.320839 36000401

[134] Jiang DS Wei X Zhang XF Liu Y Zhang Y Chen K IRF8 suppresses pathological cardiac remodelling by inhibiting calcineurin signalling. Nat Commun. (2014d) 5:3303. 10.1038/ncomms4303 24526256 PMC3929801

[135] Jiang DS Luo YX Zhang R Zhang XD Chen HZ Zhang Y Interferon regulatory factor 9 protects against cardiac hypertrophy by targeting myocardin. Hypertension. (2014c) 63:119–27. 10.1161/HYPERTENSIONAHA.113.02083 24144649

[136] Eckert M Meek SE Ball KL. A novel repressor domain is required for maximal growth inhibition by the IRF-1 tumor suppressor. J Biol Chem. (2006) 281:23092–102. 10.1074/jbc.M512589200 16679314

[137] Fragale A Gabriele L Stellacci E Borghi P Perrotti E Ilari R IFN regulatory factor-1 negatively regulates CD4+ CD25+ regulatory T cell differentiation by repressing Foxp3 expression. J Immunol. (2008) 181:1673–82. 10.4049/jimmunol.181.3.1673 18641303

[138] Patel B Bansal SS Ismahil MA Hamid T Rokosh G Mack M CCR2(+) monocyte-derived infiltrating macrophages are required for adverse cardiac remodeling during pressure overload. JACC Basic Transl Sci. (2018) 3:230–44. 10.1016/j.jacbts.2017.12.006 30062209 PMC6059350

[139] El Jamali A Freund C Rechner C Scheidereit C Dietz R Bergmann MW. Reoxygenation after severe hypoxia induces cardiomyocyte hypertrophy in vitro: activation of CREB downstream of GSK3beta. FASEB J. (2004) 18:1096–8. 10.1096/fj.03-1054fje 15155564

[140] Huggins GS Lepore JJ Greytak S Patten R Mcnamee R Aronovitz M The CREB leucine zipper regulates CREB phosphorylation, cardiomyopathy, and lethality in a transgenic model of heart failure. Am J Physiol Heart Circ Physiol. (2007) 293:H1877–82. 10.1152/ajpheart.00516.2007 17616745 PMC3911886

[141] Takaoka A Yanai H Kondo S Duncan G Negishi H Mizutani T Integral role of IRF-5 in the gene induction programme activated by toll-like receptors. Nature. (2005) 434:243–9. 10.1038/nature03308 15665823

[142] Krausgruber T Blazek K Smallie T Alzabin S Lockstone H Sahgal N IRF5 promotes inflammatory macrophage polarization and TH1-TH17 responses. Nat Immunol. (2011) 12:231–8. 10.1038/ni.1990 21240265

[143] Becher PM Hinrichs S Fluschnik N Hennigs JK Klingel K Blankenberg S Role of toll-like receptors and interferon regulatory factors in different experimental heart failure models of diverse etiology: IRF7 as novel cardiovascular stress-inducible factor. PLoS One. (2018) 13:e0193844. 10.1371/journal.pone.0193844 29538462 PMC5851607

[144] Molkentin JD Lu JR Antos CL Markham B Richardson J Robbins J A calcineurin-dependent transcriptional pathway for cardiac hypertrophy. Cell. (1998) 93:215–28. 10.1016/S0092-8674(00)81573-1 9568714 PMC4459646

[145] Badorff C Seeger FH Zeiher AM Dimmeler S. Glycogen synthase kinase 3beta inhibits myocardin-dependent transcription and hypertrophy induction through site-specific phosphorylation. Circ Res. (2005) 97:645–54. 10.1161/01.RES.0000184684.88750.FE 16141410

[146] Li S Wang J Yan Y Zhang Z Gong W Nie S. Clinical characterization and possible pathological mechanism of acute myocardial injury in COVID-19. Front Cardiovasc Med. (2022a) 9:862571. 10.3389/fcvm.2022.862571 35387441 PMC8979292

[147] Gao YD Ding M Dong X Zhang JJ Kursat Azkur A Azkur D Risk factors for severe and critically ill COVID-19 patients: a review. Allergy. (2021) 76:428–55. 10.1111/all.14657 33185910

[148] Eskandarian Boroujeni M Sekrecka A Antonczyk A Hassani S Sekrecki M Nowicka H Dysregulated interferon response and immune hyperactivation in severe COVID-19: targeting STATs as a novel therapeutic strategy. Front Immunol. (2022) 13:888897. 10.3389/fimmu.2022.888897 35663932 PMC9156796

[149] Pairo-Castineira E Clohisey S Klaric L Bretherick AD Rawlik K Pasko D Genetic mechanisms of critical illness in COVID-19. Nature. (2021) 591:92–8. 10.1038/s41586-020-03065-y 33307546

[150] Consortium W.H.O.S.T., Pan H Peto R Henao-Restrepo AM Preziosi MP Sathiyamoorthy V Repurposed antiviral drugs for COVID-19—interim WHO solidarity trial results. N Engl J Med. (2021) 384:497–511. 10.1056/NEJMoa2023184 33264556 PMC7727327

[151] Group RC. Baricitinib in patients admitted to hospital with COVID-19 (RECOVERY): a randomised, controlled, open-label, platform trial and updated meta-analysis. Lancet. (2022) 400:359–68. 10.1016/S0140-6736(22)01109-6 35908569 PMC9333998

[152] Chen HJ Tas SW De Winther MPJ. Type-I interferons in atherosclerosis. J Exp Med. (2020) 217:e20190459. 10.1084/jem.20190459 31821440 PMC7037237

[153] Voloshyna I Littlefield MJ Reiss AB. Atherosclerosis and interferon-gamma: new insights and therapeutic targets. Trends Cardiovasc Med. (2014) 24:45–51. 10.1016/j.tcm.2013.06.003 23916809 PMC3844070

[154] Elyasi A Voloshyna I Ahmed S Kasselman LJ Behbodikhah J De Leon J The role of interferon-gamma in cardiovascular disease: an update. Inflamm Res. (2020) 69:975–88. 10.1007/s00011-020-01382-6 32699989

[155] Fang L Ellims AH Beale AL Taylor AJ Murphy A Dart AM. Systemic inflammation is associated with myocardial fibrosis, diastolic dysfunction, and cardiac hypertrophy in patients with hypertrophic cardiomyopathy. Am J Transl Res. (2017) 9:5063–73. 29218105 PMC5714791

[156] Forster O Hilfiker-Kleiner D Ansari AA Sundstrom JB Libhaber E Tshani W Reversal of IFN-gamma, oxLDL and prolactin serum levels correlate with clinical improvement in patients with peripartum cardiomyopathy. Eur J Heart Fail. (2008) 10:861–8. 10.1016/j.ejheart.2008.07.005 18768352

[157] Van Ommen AM Diez Benavente E Onland-Moret NC Valstar GB Cramer MJ Rutten FH Plasma proteomic patterns show sex differences in early concentric left ventricular remodeling. Circ Heart Fail. (2023) 16:e010255. 10.1161/CIRCHEARTFAILURE.122.010255 37381923 PMC10348648

[158] Londe AC Fernandez-Ruiz R Julio PR Appenzeller S Niewold TB. Type I interferons in autoimmunity: implications in clinical phenotypes and treatment response. J Rheumatol. (2023) 50:1103–13. 10.3899/jrheum.2022-0827 37399470 PMC12841979

[159] Miric M Miskovic A Vasiljevic JD Keserovic N Pesic M. Interferon and thymic hormones in the therapy of human myocarditis and idiopathic dilated cardiomyopathy. Eur Heart J. (1995) 16(Suppl O):150–2. 10.1093/eurheartj/16.suppl_O.150 8682086

[160] Kuhl U Pauschinger M Schwimmbeck PL Seeberg B Lober C Noutsias M Interferon-beta treatment eliminates cardiotropic viruses and improves left ventricular function in patients with myocardial persistence of viral genomes and left ventricular dysfunction. Circulation. (2003) 107:2793–8. 10.1161/01.CIR.0000072766.67150.51 12771005

[161] Zimmermann O Rodewald C Radermacher M Vetter M Wiehe JM Bienek-Ziolkowski M Interferon beta-1b therapy in chronic viral dilated cardiomyopathy–is there a role for specific therapy? J Card Fail. (2010) 16:348–56. 10.1016/j.cardfail.2009.12.016 20350703

[162] Schultheiss HP Piper C Sowade O Waagstein F Kapp JF Wegscheider K Betaferon in chronic viral cardiomyopathy (BICC) trial: effects of interferon-beta treatment in patients with chronic viral cardiomyopathy. Clin Res Cardiol. (2016) 105:763–73. 10.1007/s00392-016-0986-9 27112783

[163] Maisch B Pankuweit S. Standard and etiology-directed evidence-based therapies in myocarditis: state of the art and future perspectives. Heart Fail Rev. (2013) 18:761–95. 10.1007/s10741-012-9362-7 23225133

[164] Anonymous. Available online at: https://pdf.hres.ca/dpd_pm/00049539.PDF (accessed March 16, 2024) (2024a).

[165] Anonymous. Available online at: https://health-products.canada.ca/dpd-bdpp/info?lang=eng&code=97056 (accessed March 16, 2024) (2024b).

[166] Anonymous. Available online at: https://pdf.hres.ca/dpd_pm/00073108.PDF (accessed March 16, 2024) (2024c).

[167] Sanchez GAM Reinhardt A Ramsey S Wittkowski H Hashkes PJ Berkun Y JAK1/2 Inhibition with baricitinib in the treatment of autoinflammatory interferonopathies. J Clin Invest. (2018) 128:3041–52. 10.1172/JCI98814 29649002 PMC6026004

[168] Tuttle KR Brosius FC 3rd Adler SG Kretzler M Mehta RL Tumlin JA JAK1/JAK2 inhibition by baricitinib in diabetic kidney disease: results from a phase 2 randomized controlled clinical trial. Nephrol Dial Transplant. (2018) 33:1950–9. 10.1093/ndt/gfx377 29481660 PMC6212720

[169] Thomson DW Bergamini G. Recent progress in small molecule TBK1 inhibitors: a patent review (2015–2020). Expert Opin Ther Pat. (2021) 31:785–94. 10.1080/13543776.2021.1904893 33724136

[170] Amouzegar A Chelvanambi M Filderman JN Storkus WJ Luke JJ. STING Agonists as cancer therapeutics. Cancers. (2021) 13:2695. 10.3390/cancers13112695 34070756 PMC8198217

[171] Tian Z Zeng Y Peng Y Liu J Wu F. Cancer immunotherapy strategies that target the cGAS-STING pathway. Front Immunol. (2022) 13:996663. 10.3389/fimmu.2022.996663 36353640 PMC9639746

[172] Leonard WJ Lin JX. Strategies to therapeutically modulate cytokine action. Nat Rev Drug Discov. (2023) 22:827–54. 10.1038/s41573-023-00746-x 37542128

[173] Hao Y Hao S Andersen-Nissen E Mauck WM 3rd Zheng S Butler A Integrated analysis of multimodal single-cell data. Cell. (2021) 184:3573–87-e3529. 10.1016/j.cell.2021.04.048 34062119 PMC8238499

[174] Calcagno DM Ng RP Jr Toomu A Zhang C Huang K Aguirre AD The myeloid type I interferon response to myocardial infarction begins in bone marrow and is regulated by Nrf2-activated macrophages. Sci Immunol. (2020) 5:eaaz1974. 10.1126/sciimmunol.aaz1974 32978242 PMC7808338

[175] Suryawanshi H Clancy R Morozov P Halushka MK Buyon JP Tuschl T. Cell atlas of the foetal human heart and implications for autoimmune-mediated congenital heart block. Cardiovasc Res. (2020) 116:1446–57. 10.1093/cvr/cvz257 31589297 PMC7314636

[176] Ma M Xu Y Su Y Ong SB Hu X Chai M Single-cell transcriptome analysis decipher new potential regulation mechanism of ACE2 and NPs signaling among heart failure patients infected with SARS-CoV-2. Front Cardiovasc Med. (2021) 8:628885. 10.3389/fcvm.2021.628885 33718452 PMC7952310

[177] Brauninger H Stoffers B Fitzek ADE Meissner K Aleshcheva G Schweizer M Cardiac SARS-CoV-2 infection is associated with pro-inflammatory transcriptomic alterations within the heart. Cardiovasc Res. (2022) 118:542–55. 10.1093/cvr/cvab322 34647998 PMC8803085

[178] Hua X Bao M Mo H Sun Z Xu M Chen X STING regulates the transformation of the proinflammatory macrophage phenotype by HIF1A into autoimmune myocarditis. Int Immunopharmacol. (2023) 121:110523. 10.1016/j.intimp.2023.110523 37354779

[179] Ma P Liu J Qin J Lai L Heo GS Luehmann H Expansion of pathogenic cardiac macrophages in immune checkpoint inhibitor myocarditis. Circulation. (2023) 149:48–66. 10.1161/CIRCULATIONAHA.122.062551 37746718 PMC11323830

[180] Wu Z Liang J Zhu S Liu N Zhao M Xiao F Single-cell analysis of graft-infiltrating host cells identifies caspase-1 as a potential therapeutic target for heart transplant rejection. Front Immunol. (2023a) 14:1251028. 10.3389/fimmu.2023.1251028 37781362 PMC10535112

[181] Dzimianski JV Scholte FEM Bergeron É Pegan SD. ISG15: it’s complicated. J Mol Biol. (2019) 431:4203–16. 10.1016/j.jmb.2019.03.013 30890331 PMC6746611

